# Flexoelectric and size-dependent effects on hygro-thermal vibration of variable thickness fluid-infiltrated porous metal foam nanoplates

**DOI:** 10.1016/j.heliyon.2024.e26150

**Published:** 2024-02-14

**Authors:** Thu-Huong Nguyen Thi, Van Ke Tran, Quoc Hoa Pham

**Affiliations:** a Faculty of Mechanical Engineering, Hanoi University of Industry, Hanoi, Viet Nam; b Faculty of Mechanical Engineering, Le Quy Don Technical University, Hanoi, Viet Nam; c Faculty of Engineering and Technology, Nguyen Tat Thanh University, Ho Chi Minh City, Viet Nam

**Keywords:** Nanoplates, Free vibration, Analytical solution, Flexoelectricity, Variable thickness

## Abstract

The Galerkin-Vlasov approach based on the improved first-order shear deformation theory (i-FSDT) and nonlocal elasticity theory are proposed to investigate the free vibration response of variable-thickness fluid-infiltrated porous metal foam (FPMF) nanoplates with flexoelectricity effect resting on Pasternak elastic foundation in the hygro-thermal environment. The FPMF nanoplate thickness varies according to both the length and width directions. The novelty of the present work is to consider the influence of the nonlocal's spatial variation and flexoelectric coefficients on the free vibration behavior of the nanoplates. Based on Hamilton's principle, the governing equation of FPMF nanoplate is established. The accuracy of the proposed method is checked by comparing the obtained results with those of available work in the literature. The effects of the parameters such as the flexoelectric coefficient, nonlocal coefficient, porosity coefficient, Skempton factor, temperature and moisture, thickness variation, and various boundary conditions on the natural frequency of the nanoplate are examined.

## Highlights

• The vibration response of fluid-infiltrated porous metal foam nanoplates.
• The flexoelectricity and size small effects on the free vibration of nanoplates are considered.
• The nanoplate's thickness varies in both the length and width directions.
• The nonlocal and flexoelectric coefficients vary along the thickness.
• Analytical methods for calculating non-uniform thickness nanoplate under different boundary conditions.

## 1 Introduction

Due to their favorable stiffness-to-weight ratio, lightweight materials have found extensive use in various branches of engineering. Among these materials, porous materials including metal foams, play a significant role in industries such as aerospace engineering, automotive manufacturing, and civil construction. They are highly valued for their exceptional multi-functionality, attributed to their low specific weight, efficient energy dissipation capabilities, and improved machinability. The mechanical properties of porous plates typically exhibit a gradual transition due to variations in porosity across their thickness. As a result, researchers have shown significant interest in exploring the potential of these materials [[1], [2], [3], [4]].

In recent times, nanotechnology and nanostructures have undergone remarkable advancements, and their use in micro- and nanoelectromechanical systems (MEMS and NEMS) is rapidly expanding. As a result, it is crucial to investigate the mechanical response of these nanostructures. However, classical continuum theories are inadequate for this task, as the mechanical behavior of nanostructures differs significantly from that of macrostructures. Therefore, the development of size-dependent continuum theories such as couple stress theory [[5], [6], [7]], strain gradient elasticity [8], the nonlocal elasticity [9], is essential for analyzing the mechanical behavior of nanostructures. Among many size-dependent continuum theories, the nonlocal elasticity theory of Eringen is of most interest to scientists. Aghababaei and Reddy [10] carried out the analysis of the static and vibration of plate using the order third shear deformation theory and nonlocal elastic theory. Pradhan and Phadikar [11] proposed the analytical approach relied on the nonlocal of Eringen to study vibration of nanoplates. Awrejcewicz et al. [12] introduced the geometrically nonlinear vibrations of rectangular micro/nanoplates employing the von Kármán theory and the nonlocal elasticity theory. Hashemi et al. [13] used the nonlocal theory and Mindlin plate theory to consider small scale effects on free vibration of rectangular nano-plates. Daneshmehr et al. [14] applied the nonlocal theory to consider small-scale effects on the free vibration behaviors of the functionally graded nanoplate. Fernández-Sáez et al. [15] investigated the static bending of Euler–Bernoulli beams with different boundary and load conditions based on the nonlocal theory.

Using nonlocal theory to explore piezoelectric nanostructures, Ke et al. [16] explored the thermal oscillation of nanobeams using the differential quadrature approach. Wang et al. [17] used an exact solution to investigate the electromechanical coupling behavior of nanowires. Liu et al. [18] computed the oscillation analysis of nanoplates under thermo-electro-mechanical reactions. Zang et al. [19] studied the effect of surface and nonlocal factors on the axial wave propagation of piezoelectric nanoplates. Liu et al. [20] used the differential quadrature method (DQM) to analyze the buckling of piezoelectric nanobeams subjected to thermo-electro- mechanical loading. Ke and co-workers [21] introduced the size-dependent effects on the vibration of nanoplates with different boundary conditions (BCs). Asemi et al. [22] detected the nanoscale mass of piezoelectric films using an analytical method. Mahesh [23] investigated the nonlinear static response of carbon nanotube reinforced multiphase magneto-electro-elastic plates by using the finite element method based higher order shear deformation theory of Reddy.

Nowadays, with the advancements in materials science and computer technology, scientists have discovered numerous novel phenomena in nanoscale dielectric materials. One such phenomenon is flexoelectricity. Recent studies have revealed that these effects become more pronounced in nanoscale structures. Flexoelectricity holds great promise for various applications in electronics, including semiconductor chips, sensors, and biotechnology. Due to the intriguing nature of this phenomenon, scientists, especially those in the field of mechanics, are currently highly interested in studying it. Several notable works on flexoelectricity include the following: Zhang and co-workers [24] studied the flexoelectric influence on the oscillation of nanoplates using Ritz approximate solutions. Liang et al. [25] studied the flexoelectricity effect on nanobeams using the Bernoulli–Euler beam theory. Beni [26] investigated electro-thermal buckling in a flexoelectric microbeam. Zhang et al. [27] employed Ritz's approximate solutions to study the static bending of piezoelectric nanoplates taking into account flexoelectricity. Yang and his colleagues [28] analyzed the electromechanical behavior of nanoplates with flexoelectricity using an exact solution. Liang et al. [29,30] developed an exact solution to investigate the buckling and vibration problems of piezoelectric nanowires under flexoelectricity. Using the Love's theory for thin shells and the modified flexoelectricity theory, Babadi et al. [31] studied the effect of flexoelectricity on the free vibration of functionally graded (FG) magneto-electro-elastic nanoshells. Beni [32,33] used the non-classical theory of continuum mechanics based on a strain gradient to present the free vibration and static torsion of a coupling electromechanical flexoelectric micro/nanotube. Samani et al. [34] used the Timoshenko beam model to investigate the nonlinear geometric effects of the thermal and mechanical buckling of the nano flexoelectric beam. Ghobadi et al. [35,36] proposed the Kirchhoff plate's theory and the modified flexoelectric theory to analyze the static, free vibration and nonlinear dynamic responses of a sandwich functionally graded porous nanoplates. Babadi et al. [37] developed the first-order shear deformation theory and the reformulated flexoelectric theory for analyzing the static and dynamic responses of the multilayered composite shell with simply supported and clamped boundary conditions. Amiri [38] studied the vibrations and instability analysis of fluid-conveying piezoelectric nanotubes on the basis of flexoelectricity approach. Arefi et al. [39] investigated the nonlinear vibration of the FG nano-beams relied on the nonlocal elasticity theory taking into account surface and flexoelectric effects. Arani et al. [40,41] studied the vibration of FG porous sandwich microbeam and annular nanoplate considering flexoelectric effects. The studies demonstrate that both flexoelectric and size-dependent effects have a significant impact on the mechanical response of nanostructures. Therefore, it motivates us to carry out the analysis of these phenomes of nanoplates to make sound recommendations in the fabrication of structures at the nanoscale.

Through a survey of available studies in the literature, to the best of the authors' knowledge, it is found that the study of the influence of flexoelectric and size-dependent phenomena on nanoplates in the multiphysics environment is still limited. In this study, the hygro-thermal vibration of nanoplates is examined, taking into account variations in thickness as well as the flexoelectric and size-dependent characteristics of the material. The novel of this paper is to present the study on the mechanical response of nanoplates by accounting for the flexoelectric effects, as well as the nonlocal coefficient, which varies in both the length and width directions of the nanoplates. The natural frequency of nanoplates under different boundary conditions is determined using the Galerkin-Vlasov method based on i-FSDT. The results obtained from the proposed method were compared with other available methods to demonstrate their effectiveness and accuracy. After checking the calculation method and program, some of the parameters such as the flexoelectric coefficient, nonlocal coefficient, porosity coefficient, Skempton factor, temperature and moisture, thickness variation, and boundary conditions on the natural frequency of the nanoplate are examined in detail. This study illuminates the size-dependent behavior of fluid-infiltrated porous metal foam piezoelectric nanostructures with varying thicknesses, opening up possibilities for their application in the development of innovative nanoplate based sensors for NEMS. Moreover, it holds promise for utilization in energy-absorbing components for diverse applications such as solar cells, mobile phones, medical equipment, and aerospace. Although there are certain advantages, however, the limitation of this study is that it only considers the problem of linear vibrations and the plate shape structure must be symmetrical due to the limitation of the analytical method. Next to the overview, theoretical formulas are presented in depth in Section 2. The technique of using the method will be presented in Section 3. Section 4 consists of two main sections, including (1) verifying the accuracy of the proposed approach and (2) providing novel numerical results on the free vibrations of nanoplates under the influence of input parameters. This study's innovative results and contributions would like to be included in the conclusion.

## 2 Theoretical formulations

### 2.1 Mechanical model and materials

**Fig. 1:**
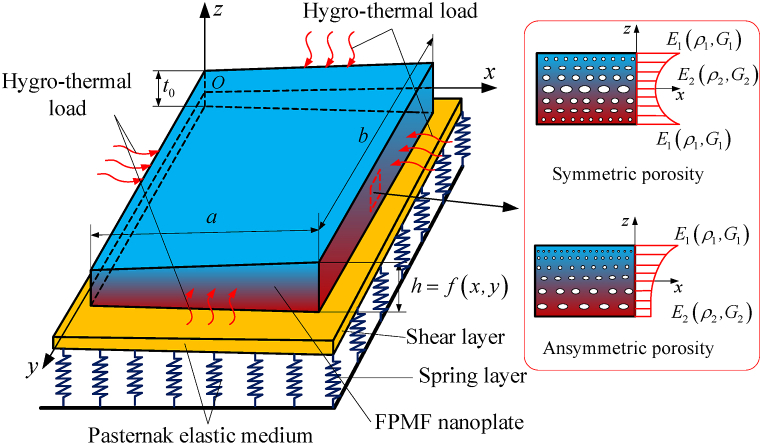
Model of the variable thickness FPMF nanoplate resting on Pasternak elastic foundation.

Considering a rectangular variable thickness FPMF nanoplate with dimensions: length a, width b, and thickness h=h(x,y). The whole nanoplate is resting on Pasternak elastic foundation with two stiffness parameters $k_{w},k_{s}$ as shown in Fig. 1. Material characteristics, including Young's modulus, shear modulus, and mass density that vary according to the laws of symmetric and asymmetric porosity distributions [42], can be expressed by Eq. (1)- (3):

Symmetric porosity distribution (Type 1)

(1) $$\left\{ \begin{array}{c} E\left(z\right)=E_{1}\left(1-\lambda_{0}\;\cos\left(\frac{\pi z}{h\left(x,y\right)}\right)\right);G\left(z\right)=G_{1}\left(1-\lambda_{0}\;\cos\left(\frac{\pi z}{h\left(x,y\right)}\right)\right);\\ \rho \left(z\right)=\rho_{1}\left(1-\sqrt{1-\lambda_{0}}\cos\left(\frac{\pi z}{h\left(x,y\right)}\right)\right);\; \end{array}\right.$$

in which $\lambda_{0}$ is a porosity coefficient represented by

(2) $$\lambda_{0}=1-E_{2}/E_{1}=1-G_{2}/G_{1};\;\left(0<\lambda_{0}<1\right)$$

where $E_{1}$, $G_{1}$ and $\rho_{1}$ are respectively the maximum value of Young's modulus, the modulus of shear, and the mass density of the porous metal foam, respectively; and $E_{2}$ and $G_{2}$ are respectively the minimum value of the aforementioned variants. In the present work, the ratio of Poisson is assumed constant.

Asymmetric porosity distribution (Type 2)

(3) $$\left\{ \begin{array}{c} E\left(z\right)=E_{1}\left(1-\lambda_{0}\;\cos\left(\frac{\pi z}{2h\left(x,y\right)}+\frac{\pi }{4}\right)\right);G\left(z\right)=G_{1}\left(1-\lambda_{0}\;\cos\left(\frac{\pi z}{2h\left(x,y\right)}+\frac{\pi }{4}\right)\right);\\ \rho \left(z\right)=\rho_{1}\left(1-\sqrt{1-\lambda_{0}}\cos\left(\frac{\pi z}{2h\left(x,y\right)}+\frac{\pi }{4}\right)\right);\; \end{array}\right.$$

**Fig. 2:**
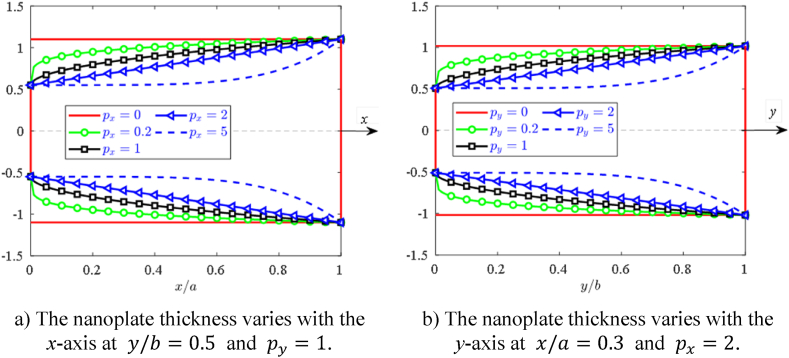
The nanoplate thickness varies with the *x*-axis (a) and *y*-axis (b).

The nanoplate thickness h=h(x,y) change in both directions following formula [43]:

(4) $$h\left(x,y\right)=t_{0}\left(1+\gamma_{x}{\left(\frac{x}{a}\right)}^{p_{x}}\right)\left(1+\gamma_{y}{\left(\frac{y}{b}\right)}^{p_{y}}\right)$$

where $t_{0}$ is the base thickness; $p_{x},p_{y},\gamma_{x}\text{,}$ and $\gamma_{y}$ represent the nanoplate thickness control parameters in the *x*- and *y*-axis. With $t_{0}=0.2\;nm;\;\gamma_{x}=\gamma_{y}=2$, the nanoplate thickness via the change of $p_{x},p_{y}$ are presented in Fig. 2a–b.

### 2.2 The combination of nonlocal elasticity theory and flexoelectric effect

Internal pores in porous configurations are typically filled with a fluid such as gas or liquid. Due to the existence of this freely moving fluid throughout the porous structure, its mechanical response and properties could be modified and tailored in the desired manner [44]. Biot's constitutive equations comprehensively describe the behavior of porous media. These equations were obtained according to two main assumptions [44].

(1). An increase of pore pressure induces a dilation of pore.
(2). Compression of the pore causes a rise of pore pressure

According to the Biot's poroelasticity theory [44] and nonlocal elasticity model ([45,46]) combine the flexoelectricic effect ([47,48,49]), the linear constitutive equations describing stress‐strain relationships for a fluid‐filled porous material can be expressed as Eqs. (5), (6), (7), (8).

(5) $${\left(1-\eta \nabla^{2}\right)\sigma }_{ij}=2G\varepsilon_{ij}+\frac{2Gv_{u}}{1-v_{u}}\epsilon \delta_{ij}-\tilde{M}\left(\vartheta -\alpha \epsilon \right)\alpha \delta_{ij}-e_{kij}E_{k,}$$

(6) $$\left(1-\eta \nabla^{2}\right)\tau_{ijm}=-f_{ijkl}E_{k}$$

(7) $$\left(1-\eta \nabla^{2}\right)\Phi_{i}=e_{ijk}\varepsilon_{jk}+\kappa_{ij}E_{k}+f_{ijkl}\varepsilon_{ij,k}$$

(8) $$\tilde{M}=\frac{2G\left(v_{u}-v\right)}{\alpha^{2}\left(1-2v_{u}\right)\left(1-2v\right)};v_{u}=\frac{v+\frac{\alpha \beta \left(1-2v\right)}{3}}{1-\frac{\alpha \beta \left(1-2v\right)}{3}}\text{.}$$

where $c_{ijkl}$ are the material characteristics of the elastic; $E_{k}$ is an electric field component; $\sigma_{ij}\text{,}$ $\varepsilon_{ij}$ and $e_{kij}$ represent the stress, strain components and piezoelectric constant tensor, respectively; $\Phi_{i}$ is the electric displacement vector; $\tau_{ijm}$ is the higher-level stress tensor, and $\kappa_{ij}$ is the permittivity constant component. η and $f_{ijkl}$ represents the nonlocal coefficient in nanostructures and the flexoelectric coefficient; $\tilde{M}$ denotes the Biot modulus, which shows the increase in the amount of fluid; $v_{u}$ is the undrained Poisson ratio, and ϵ represents the volumetric strain; $\delta_{ij}$ is the Kronecker delta function; ϑ is the variation in the fluid volume component inside the porosity; α is the Biot factor of effective stress 0<α<1, and β is the Skempton parameter.

### 2.3 The improved first-order shear deformation plate theory

The displacement fields along the three coordinate axes, i. e, the $O_{x}$, $O_{y}$ and $O_{z}$ axes at any point are represented as follows [46,50]:

(9) $$\left\{ \begin{array}{c} u_{x}\left(x,y,z,t\right)=u_{0}\left(x,y,t\right)+z\varphi_{x}\\ u_{y}\left(x,y,z,t\right)=v_{0}\left(x,y,t\right)+z\varphi_{y}\\ u_{z}\left(x,y,z,t\right)=w_{0}\; \end{array}\right.$$

where $u_{0},v_{0},\varphi_{x},\varphi_{y}\text{,}$ and $w_{0}$ are five unknown displacement components.

The linear strain fields according to the displacement field Eq. (9) are determined as Eqs. (10), (11) below:

(10) $$\varepsilon_{xx}=u_{0,x}+z\varphi_{x,x};\varepsilon_{yy}=v_{0,y}+z\varphi_{y,y};\varepsilon_{xy}=u_{0,y}+v_{0,x}+z\left(\varphi_{x,y}+\varphi_{y,x}\right)\text{;}$$

(11) $$\gamma_{xz}=\varphi_{x}+w_{0,x};\gamma_{yz}=\varphi_{y}+w_{0,y}\text{.}$$

As well known that the FSDT cannot compute transverse shear stress equal to zero at the top and bottom surfaces of nanoplates. To overcome the above disadvantages of the traditional FSDT theory, this study proposes a formula which depends on the thickness z, called i-FSDT [51], given as Eq. (12) follows:

(12) $$\gamma_{xz}^{i}=g\left(z\right)\gamma_{xz};\gamma_{yz}^{i}=g\left(z\right)\gamma_{yz}$$

where g(z) is the function of transverse shear stress the distribution through the plate's thickness and g(z) is selected by Eq. (13):

(13) $$g\left(z\right)=\frac{5}{3.89}\left(1-{\left(\frac{z}{h}\right)}^{2}\right)\text{;}$$

The stress-strain relations, or constitutive interactions, of elastic materials are written as Eq. (14) bellow:

(14) $$\left\{\begin{array}{c} \sigma_{xx}\\ \sigma_{yy}\\ \begin{array}{c} \sigma_{xy} \end{array} \end{array}\right\}=\left[\begin{array}{ccc} C_{11} & C_{12} & 0\\ C_{12} & C_{22} & 0\\ 0 & 0 & C_{66} \end{array}\right]\left\{\begin{array}{c} \varepsilon_{xx}\\ \varepsilon_{yy}\\ \varepsilon_{xy} \end{array}\right\};\left\{\begin{array}{c} \sigma_{xz}\\ \sigma_{yz} \end{array}\right\}=\left[\begin{array}{cc} C_{55} & 0\\ 0 & C_{44} \end{array}\right]\left\{\begin{array}{c} \gamma_{xz}^{i}\\ \gamma_{yz}^{i} \end{array}\right\}\text{.}$$

The below Eqs. (15), (16) is employed to take the constitutive constant components $C_{kl}$ [52]:

(15) $$C_{11}=C_{22}=\frac{E\left(z\right)}{2\left(1+v\right)}\left(\frac{2}{1-v_{u}^{2}}\right)\left(1+v_{u}+\frac{\left(v_{u}-v\right)\left(1+v_{u}\right)}{1-2v}\left(1-\frac{S_{2}}{S_{1}}\right)\right);\;C_{12}=\frac{E\left(z\right)}{2\left(1+v\right)}\left(\frac{2}{1-v_{u}^{2}}\right)\left(\left(1+v_{u}\right)v_{u}+\frac{\left(v_{u}-v\right)\left(1+v_{u}\right)}{1-2v}\left(1-\frac{S_{2}}{S_{1}}\right)\right);\;C_{66}=C_{55}=C_{44}=\frac{E\left(z\right)}{2\left(1+v\right)}\text{;}$$

(16) $$S_{1}=\frac{E\left(z\right)}{1+v}\left(1+\frac{v_{u}}{1-2v_{u}}+\frac{\left(v_{u}-v\right)\left(1+v_{u}\right)}{1-2v}\right),S_{2}=\frac{E\left(z\right)}{1+v}\left(\frac{v_{u}}{1-2v_{u}}+\frac{\left(v_{u}-v\right)\left(1+v_{u}\right)}{1-2v}\right)$$

Due to the influence of the flexoelectricity, it is necessary to take into account the strain gradient, which has the following expression (see Eq. (17)):

(17) $$\chi_{xxz}=\varepsilon_{xx,z}=\varphi_{x,x};\chi_{yyz}=\varepsilon_{yy,z}=\varphi_{y,y}$$

Assume that the electric field is along the *z*-direction only [47]. According to Eqs. (5), (6), (7), the constitutive equation of FPMF nanoplates with flexoelectricity effect can be rewritten as Eqs. (18a-d) and (19):

(18a) $$\left(1-\eta \nabla^{2}\right)\sigma_{xx}=C_{11}\varepsilon_{xx}+C_{12}\varepsilon_{yy}-e_{31}E_{z}$$

(18b) $$\left(1-\eta \nabla^{2}\right)\sigma_{yy}=C_{12}\varepsilon_{xx}+C_{22}\varepsilon_{yy}-e_{31}E_{z}$$

(18c) $$\left(1-\eta \nabla^{2}\right)\sigma_{xy}=C_{66}\varepsilon_{xy};\left(1-\eta \nabla^{2}\right)\sigma_{xz}=C_{55}\varepsilon_{xz};\left(1-\eta \nabla^{2}\right)\sigma_{yz}=C_{44}\varepsilon_{yz}$$

(18d) $$\left(1-\eta \nabla^{2}\right)\tau_{xxz}=-f_{14}H_{z};\;\left(1-\eta \nabla^{2}\right)\tau_{yyz}=-f_{14}E_{z}\text{;}$$

(19) $$\left(1-\eta \nabla^{2}\right)\Phi_{z}=e_{31}\left(\varepsilon_{xx}+\varepsilon_{yy}\right)+\kappa_{33}E_{z}+f_{14}\left(\chi_{xxz}+\chi_{yyz}\right)\text{.}$$

where $f_{14}=f_{3113}$ and $f_{14}=f_{3223}$ are convenience variables [49,53]. In the absence of free electric charges, the electric displacement should satisfy the Gaussian law in electrostatics as Eq. (20).

(20) $$\Phi_{z,z}=0$$

Under the open-circuit condition, the electric displacement on the surface is equal to zero. Hence, the internal electric field can be obtained from Eq. (21) as bellow:

(21) $$E_{z}=-\frac{e_{31}}{\kappa_{33}}\left(u_{0,x}+v_{0,y}\right)-\left(\frac{e_{31}z}{\kappa_{33}}+\frac{f_{14}}{\kappa_{33}}\right)\left(\varphi_{x,x}+\varphi_{y,y}\right)$$

In previous studies on piezoelectric nanostructures, the nonlocal and flexoelectric coefficients were often assumed to be constant throughout the material. However, this assumption is not valid for materials with variable mechanical properties. Therefore, to obtain a more accurate calculation of the natural frequency of an FPMF nanoplate, it is necessary to consider that the nonlocal and flexoelectric coefficients vary with the same law as the elastic modulus E(z). Therefore, the nonlocal and flexoelectric coefficients are expressed as Eqs. (22), (23) as follows

Symmetric porosity distribution (Type 1):

(22) $$\eta =\eta_{1}\left(1-\lambda_{0}\;\cos\left(\frac{\pi z}{h\left(x,y\right)}\right)\right);f_{14}=f_{014}\left(1-\lambda_{0}\;\cos\left(\frac{\pi z}{h\left(x,y\right)}\right)\right)\text{;}$$

**Fig. 3:**
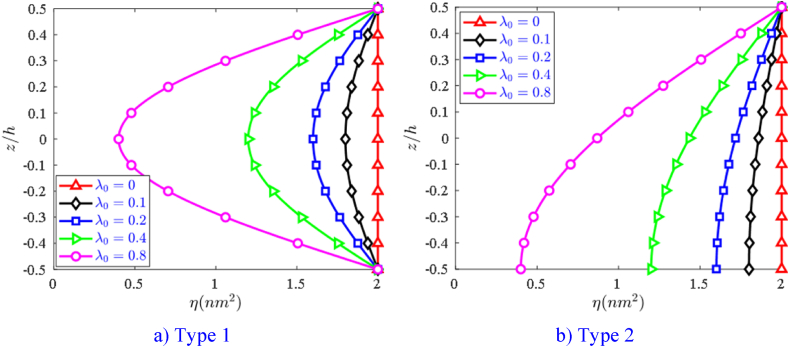
The nonlocal coefficient η(z) via the nanoplate thickness and porosity coefficient $\lambda_{0}$.

Asymmetric porosity distribution (Type 2):

(23) $$\eta =\eta_{1}\left(1-\lambda_{0}\;\cos\left(\frac{\pi z}{2h\left(x,y\right)}+\frac{\pi }{4}\right)\right);f_{14}=f_{014}\left(1-\lambda_{0}\;\cos\left(\frac{\pi z}{2h\left(x,y\right)}+\frac{\pi }{4}\right)\right)\text{.}$$

where: $\eta_{1},f_{014}$ are the initial values of the nonlocal and flexoelectricity coefficients, respectively. The assumption of the above change is a new point of this study. However, experimental and molecular simulation studies need to be performed to accurately determine these coefficients. The nonlocal coefficient η(z) in two cases of porous distribution laws is also drawn in Fig. 3a–b with $\eta_{1}=2{nm}^{2}$ and various porosity coefficient $\lambda_{0}\text{.}$.

### 2.4 Equations of motion

The motion equation of variable thickness fluid-infiltrated porous metal foam piezoelectric nanoplate is derived from Hamilton's principle as follows [[54], [55], [56], [57]]:

(24) $$\delta \Pi =\int_{t_{1}}^{t_{2}}\delta \left(U+U_{f}+V-K\right)dt=0$$

where $U,U_{f},V$ and K denote strain energy, Pasternak elastic foundation strain energy, potential energy, and kinetic energy, respectively.

The total strain energy U is:

(25) $$U=\frac{1}{2}\int_{S}\left(N_{x}u_{0,x}+M_{x}\varphi_{x,x}+Q_{x}\left(\varphi_{x}+w_{0,x}\right)+N_{y}v_{0,y}+M_{y}\varphi_{y,y}+Q_{y}\left(\varphi_{y}+w_{0,y}\right)+N_{xy}\left(u_{0,y}+v_{0,x}\right)+M_{xy}\left(\varphi_{x,y}+\varphi_{y,x}\right)+L_{xxz}\varphi_{x,x}+L_{yyz}\varphi_{y,y}\right)dS$$

in which $N_{i},M_{i},Q_{i}$ and $L_{i}$ are the local stress resultants which are determined by

(26a) $$\left\{\begin{array}{c} N_{xx}\\ N_{yy}\\ N_{xy} \end{array}\right\}=\int_{-0.5h}^{+0.5h}\left\{\begin{array}{c} \sigma_{xx}\\ \sigma_{yy}\\ \sigma_{xy} \end{array}\right\}dz;\left\{\begin{array}{c} M_{xx}\\ M_{yy}\\ M_{xy} \end{array}\right\}=\int_{-0.5h}^{+0.5h}\left\{\begin{array}{c} \sigma_{xx}\\ \sigma_{yy}\\ \sigma_{xy} \end{array}\right\}zdz$$

(26b) $$\left\{\begin{array}{c} Q_{xz}\\ Q_{yz} \end{array}\right\}=\int_{-0.5h}^{+0.5h}\left\{\begin{array}{c} \sigma_{xz}\\ \sigma_{yz} \end{array}\right\}dz;\left\{\begin{array}{c} L_{xxz}\\ L_{yyz} \end{array}\right\}=\int_{-0.5h}^{+0.5h}\left\{\begin{array}{c} \tau_{xxz}\\ \tau_{yyz} \end{array}\right\}dz$$

By replacing Eqs. (18a), (18b), (18c), (18d), (21) into Eq. (26a), (26b) and integrating through the thickness, one obtains:

(27a) $$\left\{\begin{array}{c} N_{x}\\ N_{y}\\ N_{xy}\\ M_{x}\\ M_{y}\\ M_{xy} \end{array}\right\}-\eta \left(z\right)\nabla^{2}\left\{\begin{array}{c} N_{x}\\ N_{y}\\ N_{xy}\\ M_{x}\\ M_{y}\\ M_{xy} \end{array}\right\}=\left[\begin{array}{cccccc} A_{11} & A_{12} & 0 & B_{11} & B_{12} & 0\\ A_{12} & A_{22} & 0 & B_{12} & B_{22} & 0\\ 0 & 0 & A_{66} & 0 & 0 & B_{66}\\ {\bar{B}}_{11} & {\bar{B}}_{12} & 0 & F_{11} & F_{12} & 0\\ {\bar{B}}_{12} & {\bar{B}}_{22} & 0 & F_{12} & F_{22} & 0\\ 0 & 0 & {\bar{B}}_{66} & 0 & 0 & F_{66} \end{array}\right]\left\{\begin{array}{c} u_{0,x}\\ v_{0,y}\\ u_{0,y}+v_{0,x}\\ \varphi_{x,x}\\ \varphi_{y,y}\\ \varphi_{x,y}+\varphi_{y,x} \end{array}\right\}\text{;}$$

(27b) $$\left\{\begin{array}{c} Q_{xz}\\ Q_{yz} \end{array}\right\}-\eta {\left(z\right)\nabla }^{2}\left\{\begin{array}{c} Q_{xz}\\ Q_{yz} \end{array}\right\}=\left[\begin{array}{cc} A_{44}^{s} & 0\\ 0 & A_{55}^{s} \end{array}\right]\left\{\begin{array}{c} \varphi_{x}+w_{0,x}\\ \varphi_{y}+w_{0,y} \end{array}\right\}\text{;}$$

(27c) $$\left\{\begin{array}{c} L_{xxz}\\ L_{yyz} \end{array}\right\}-\eta \left(z\right)\nabla^{2}\left\{\begin{array}{c} L_{xxz}\\ L_{yyz} \end{array}\right\}=\left[\begin{array}{cc} S_{11} & S_{12}\\ S_{11} & S_{12} \end{array}\right]\left\{\begin{array}{c} u_{0,x}+v_{0,y}\\ \varphi_{x,x}+\varphi_{y,y} \end{array}\right\}\text{.}$$

in which, the material stiffness coefficients $A_{ij},B_{ij},{\bar{B}}_{ij},F_{ij},A_{ij}^{s},S_{ij}$ are expressed as Eq. (28a), (28b), (28c), (28d), (28e), (28f) below

(28a) $$A_{11}=\int_{-\frac{h}{2}}^{\frac{h}{2}}\left(C_{11}+\frac{e_{31}^{2}}{\kappa_{33}}\right)dz,A_{12}=\int_{-\frac{h}{2}}^{\frac{h}{2}}\left(C_{12}+\frac{e_{31}^{2}}{\kappa_{33}}\right)dz,A_{22}=\int_{-\frac{h}{2}}^{\frac{h}{2}}\left(C_{22}+\frac{e_{31}^{2}}{\kappa_{33}}\right)dz,A_{66}=\int_{-\frac{h}{2}}^{\frac{h}{2}}C_{66}dz\text{,}$$

(28b) $$B_{11}=\int_{-\frac{h}{2}}^{\frac{h}{2}}\left(C_{11}z+\frac{e_{31}^{2}z}{\kappa_{33}}+\frac{e_{31}f_{14}}{\kappa_{33}}\right)dz,B_{12}=\int_{-\frac{h}{2}}^{\frac{h}{2}}\left(C_{12}z+\frac{e_{31}^{2}z}{\kappa_{33}}+\frac{e_{31}f_{14}}{\kappa_{33}}\right)dz,B_{22}=\int_{-\frac{h}{2}}^{\frac{h}{2}}\left(C_{22}z+\frac{e_{31}^{2}z}{\kappa_{33}}+\frac{e_{31}f_{14}}{\kappa_{33}}\right)dz,B_{66}=\int_{-\frac{h}{2}}^{\frac{h}{2}}C_{66}zdz\text{,}$$

(28c) $${\bar{B}}_{11}=\int_{-\frac{h}{2}}^{\frac{h}{2}}\left(C_{11}+\frac{e_{31}^{2}}{\kappa_{33}}\right)zdz,{\bar{B}}_{12}=\int_{-\frac{h}{2}}^{\frac{h}{2}}\left(C_{12}+\frac{e_{31}^{2}}{\kappa_{33}}\right)zdz,{\bar{B}}_{22}=\int_{-\frac{h}{2}}^{\frac{h}{2}}\left(C_{22}+\frac{e_{31}^{2}}{\kappa_{33}}\right)zdz,{\bar{B}}_{66}=\int_{-\frac{h}{2}}^{\frac{h}{2}}C_{66}zdz\text{,}$$

(28d) $$F_{11}=\int_{-\frac{h}{2}}^{\frac{h}{2}}\left(C_{11}z^{2}+\frac{e_{31}^{2}z^{2}}{\kappa_{33}}+\frac{e_{31}f_{14}z}{\kappa_{33}}\right)dz,F_{12}=\int_{-\frac{h}{2}}^{\frac{h}{2}}\left(C_{12}z^{2}+\frac{e_{31}^{2}z^{2}}{\kappa_{33}}+\frac{e_{31}f_{14}z}{\kappa_{33}}\right)dz,F_{22}=\int_{-\frac{h}{2}}^{\frac{h}{2}}\left(C_{22}z+\frac{e_{31}^{2}z^{2}}{\kappa_{33}}+\frac{e_{31}f_{14}z}{\kappa_{33}}\right)dz,F_{66}=\int_{-\frac{h}{2}}^{\frac{h}{2}}C_{66}z^{2}dz\text{,}$$

(28e) $$A_{44}^{s}=\int_{\frac{-h}{2}}^{\frac{h}{2}}C_{44}g\left(z\right)dz;A_{55}^{s}=\int_{\frac{-h}{2}}^{\frac{h}{2}}C_{55}g\left(z\right)dz\text{,}$$

(28f) $$S_{11}=\int_{\frac{-h}{2}}^{\frac{h}{2}}\frac{e_{31}f_{14}}{\kappa_{33}}dz,S_{12}=\int_{\frac{-h}{2}}^{\frac{h}{2}}\frac{e_{31}f_{14}z+f_{14}^{2}}{\kappa_{33}}dz\text{,}$$

The Pasternak elastic foundation strain energy $U_{f}$ is defined by Refs. [58,59]:

(29) $$U_{f}=\frac{1}{2}\int_{S}\left(k_{w}w_{0}^{2}+k_{s}{\left(w_{0,x}\right)}^{2}+k_{s}{\left(w_{0,y}\right)}^{2}\right)dS$$

The definition of potential energy V is given as follows [60]:

(30) $$V=\frac{1}{2}\int_{S}\left(\left(N_{x}^{T}+N_{x}^{C}\right){\left(w_{0,x}\right)}^{2}+\left(N_{y}^{T}+N_{y}^{C}\right){\left(w_{0,y}\right)}^{2}\right)dS$$

where $\left(N_{x}^{T},N_{y}^{T}\right)$, and $\left(N_{x}^{C},N_{y}^{C}\right)$ are forces in the horizontal plane due to thermal, and moisture reactions ([61,62]). These components are determined by Eqs. (31), (32).

(31) $$N_{x}^{T}=N_{y}^{T}=N^{T}=-\int_{-\frac{h\left(x,y\right)}{2}}^{\frac{h\left(x,y\right)}{2}}\frac{E\left(z\right)}{1-v}\bar{\alpha }\left(z\right)\left(T\left(x,y,z\right)-T_{0}\right)dz$$

(32) $$N_{x}^{C}=N_{y}^{C}=N^{C}=-\int_{-\frac{h\left(x,y\right)}{2}}^{\frac{h\left(x,y\right)}{2}}\frac{E\left(z\right)}{1-v}\bar{\beta }\left(z\right)\left(C\left(x,y,z\right)-C_{0}\right)dz$$

The linear relation of temperature and moisture [61] to the thickness of the fluid-infiltrated porous metal foam piezoelectric nanoplate is expressed as follows (see Eq. (33)):

(33) $$T\left(x,y,z\right)=T_{m}+\Delta T\left(\frac{1}{2}+\frac{z}{h\left(x,y\right)}\right);C\left(x,y,z\right)=C_{m}+\Delta C\left(\frac{1}{2}+\frac{z}{h\left(x,y\right)}\right)\text{.}$$

in which $\Delta T=T_{c}-T_{m};\Delta C=C_{c}-C_{m}$ are temperature and moisture changes.

The kinetic energy K is defined by

(34) $$K=\frac{1}{2}\int_{S}\int_{-h/2}^{h/2}\rho \left(z\right)\left({\left({\dot{u}}_{x}\right)}^{2}+{\left({\dot{u}}_{y}\right)}^{2}+{\left({\dot{u}}_{z}\right)}^{2}\right)dzdS=\frac{1}{2}\int_{S}\int_{-h/2}^{h/2}\rho \left(z\right)\left({\left({\dot{u}}_{0}+z{\dot{\varphi }}_{x}\right)}^{2}+{\left({\dot{v}}_{0}+z{\dot{\varphi }}_{y}\right)}^{2}+{\left({\dot{w}}_{0}\right)}^{2}\right)dzdS$$

The motion equations may be found by substituting Eqs. (25), (27a), (27b), (27c), (29), (30), (34) into Eq. (24) can be given as:

(35) $$N_{x,x}+N_{xy,y}=I_{0}{\ddot{u}}_{0}+I_{1}{\ddot{\varphi }}_{x}-\nabla^{2}\left(J_{0}{\ddot{u}}_{0}+J_{1}{\ddot{\varphi }}_{x}\right)$$

(36) $$N_{xy,x}+N_{y,y}=I_{0}{\ddot{v}}_{0}+I_{1}{\ddot{\varphi }}_{y}-\nabla^{2}\left(J_{0}{\ddot{v}}_{0}+J_{1}{\ddot{\varphi }}_{y}\right)$$

(37) $$M_{x,x}+M_{xy,y}-Q_{xz}+L_{xxz,x}=I_{1}{\ddot{u}}_{0}+I_{2}{\ddot{\varphi }}_{x}-\nabla^{2}\left(J_{1}{\ddot{u}}_{0}+J_{2}{\ddot{\varphi }}_{x}\right)$$

(38) $$M_{xy,x}+M_{y,y}-Q_{yz}+L_{yyz,y}=I_{1}{\ddot{v}}_{0}+I_{2}{\ddot{\varphi }}_{y}-\nabla^{2}\left(J_{1}{\ddot{v}}_{0}+J_{2}{\ddot{\varphi }}_{y}\right)$$

(39) $$Q_{xz,x}+Q_{yz,y}=k_{w}w_{0}-\left(k_{s}+N^{T}+N^{C}+k_{\mu w}\right)\nabla^{2}w_{0}+\nabla^{2}\left(k_{s\mu }+N^{\mu T}+N^{\mu C}\right)\nabla^{2}w_{0}+I_{0}{\ddot{w}}_{0}-\nabla^{2}J_{0}{\ddot{w}}_{0}$$

Because the effective nonlocal coefficient is assumed to change smoothly by the thickness of the variable thickness fluid-infiltrated porous metal foam piezoelectric nanoplates, the parameters $I_{0},I_{1},I_{2}$ and $J_{0},J_{1},J_{2}$ are calculated as Eqs. (40), (41), (42), (43), (44) bellow:

(40) $$\left(I_{0},I_{1},I_{2}\right)=\int_{-h\left(x,y\right)/2}^{h\left(x,y\right)/2}\left(1,z,z^{2}\right)\rho \left(z\right)dz$$

(41) $$\left(J_{0},J_{1},J_{2}\right)=\int_{-h\left(x,y\right)/2}^{h\left(x,y\right)/2}\left(1,z,z^{2}\right)\rho \left(z\right)\eta \left(z\right)dz$$

where $k_{\mu w},k_{s\mu },N^{\mu T},N^{\mu C}$ are determined by

(42) $$k_{\mu w}=\eta \left(-h\left(x,y\right)/2\right)k_{w},k_{\mu s}=\eta \left(-h\left(x,y\right)/2\right)k_{s}$$

(43) $$N^{\mu T}=-\int_{-\frac{h\left(x,y\right)}{2}}^{\frac{h\left(x,y\right)}{2}}\frac{E\left(z\right)}{1-v}\bar{\alpha }\left(z\right)\eta \left(z\right)\left(T\left(x,y,z\right)-T_{0}\right)dz$$

(44) $$N^{\mu C}=-\int_{-\frac{h\left(x,y\right)}{2}}^{\frac{h\left(x,y\right)}{2}}\frac{E\left(z\right)}{1-v}\bar{\beta }\left(z\right)\eta \left(z\right)\left(C\left(x,y,z\right)-C_{0}\right)dz$$

Then, the motion equation in terms of displacements by inserting Eq. (27a), (27b), (27c) into Eqs. (35), (36), (37), (38), (39) as follows:

(45) $${\left(A_{11}u_{0,x}+A_{12}v_{0,y}+B_{11}\varphi_{x,x}+B_{12}\varphi_{y,y}\right)}_{,x}+{\left(A_{66}\left(u_{0,y}+v_{0,x}\right)+B_{66}\left(\varphi_{x,y}+\varphi_{y,x}\right)\right)}_{,y}-I_{0}{\ddot{u}}_{0}-I_{1}{\ddot{\varphi }}_{x}+{\left(J_{0}{\ddot{u}}_{0}+J_{1}{\ddot{\varphi }}_{x}\right)}_{,xx}+{\left(J_{0}{\ddot{u}}_{0}+J_{1}{\ddot{\varphi }}_{x}\right)}_{,yy}=0\text{,}$$

(46) $${\left(A_{66}\left(u_{0,y}+v_{0,x}\right)+B_{66}\left(\varphi_{x,y}+\varphi_{y,x}\right)\right)}_{,x}+{\left(A_{12}u_{0,x}+A_{22}v_{0,y}+B_{12}\varphi_{x,x}+B_{22}\varphi_{y,y}\right)}_{,y}-I_{0}{\ddot{v}}_{0}-I_{1}{\ddot{\varphi }}_{y}+{\left(J_{0}{\ddot{v}}_{0}+J_{1}{\ddot{\varphi }}_{y}\right)}_{,xx}+{\left(J_{0}v_{0}+J_{1}{\ddot{\varphi }}_{y}\right)}_{,yy}=0\text{,}$$

(47) $${\left({\bar{B}}_{11}u_{0,x}+{\bar{B}}_{12}v_{0,y}+F_{11}\varphi_{x,x}+F_{12}\varphi_{y,y}\right)}_{,x}+{\left({\bar{B}}_{66}\left(u_{0,y}+v_{0,x}\right)+F_{66}\left(\varphi_{x,y}+\varphi_{y,x}\right)\right)}_{,y}-A_{44}^{s}\left(\varphi_{x}+w_{0,x}\right)+{\left(S_{11}\left(u_{0,x}+v_{0,y}\right)+S_{12}\left(\varphi_{x,x}+\varphi_{y,y}\right)\right)}_{,x}-I_{1}{\ddot{u}}_{0}-I_{2}{\ddot{\varphi }}_{x}+{\left(J_{1}{\ddot{u}}_{0}+J_{2}{\ddot{\varphi }}_{x}\right)}_{,xx}+{\left(J_{1}{\ddot{u}}_{0}+J_{2}{\ddot{\varphi }}_{x}\right)}_{,yy}=0\text{,}$$

(48) $${\left({\bar{B}}_{66}\left(u_{0,y}+v_{0,x}\right)+F_{66}\left(\varphi_{x,y}+\varphi_{y,x}\right)\right)}_{,x}+{\left({\bar{B}}_{12}u_{0,x}+{\bar{B}}_{22}v_{0,y}+F_{12}\varphi_{x,x}+F_{22}\varphi_{y,y}\right)}_{,y}-A_{55}^{s}\left(\varphi_{y}+w_{0,y}\right)+{\left(S_{11}\left(u_{0,x}+v_{0,y}\right)+S_{12}\left(\varphi_{x,x}+\varphi_{y,y}\right)\right)}_{,y}-I_{1}{\ddot{v}}_{0}-I_{2}{\ddot{\varphi }}_{y}+{\left(J_{1}{\ddot{v}}_{0}+J_{2}{\ddot{\varphi }}_{y}\right)}_{,xx}+{\left(J_{1}v_{0}+J_{2}{\ddot{\varphi }}_{y}\right)}_{,yy}=0\text{,}$$

(49) $${\left(A_{44}^{s}\left(\varphi_{x}+w_{0,x}\right)\right)}_{,x}+{\left(A_{55}^{s}\left(\varphi_{y}+w_{0,y}\right)\right)}_{,y}-k_{w}w_{0}+\left(k_{s}+N^{T}+N^{C}+k_{\mu w}\right)\left(w_{0,xx}+w_{0,yy}\right)-{\left(\left(k_{s\mu }+N^{\mu 0}+N^{\mu T}+N^{\mu C}\right)\left(w_{0,xx}+w_{0,yy}\right)\right)}_{,xx}-{\left(\left(k_{s\mu }+N^{\mu 0}+N^{\mu T}+N^{\mu C}\right)\nabla^{2}w_{0}\right)}_{,yy}-I_{0}{\ddot{w}}_{0}+{\left(J_{0}{\ddot{w}}_{0}\right)}_{,xx}+{\left(J_{0}{\ddot{w}}_{0}\right)}_{,yy}=0$$

## 3 Galerkin-Vlasov approach

In this work, the Galerkin-Vlasov approach [63] is used to solve for the variable thickness FPMF nanoplates under various boundary conditions. The following are the boundary criteria that must be met for solving the motion equation (see Eqs. (50), (51)).

For fully clamped (C):

(50) $$u_{0}=v_{0}=w_{0}=\varphi_{x}=\varphi_{y}=0\;\text{at}\;x=0,a\;\text{and}\;y=0,b$$

For simply supported (S):

(51) $$u_{0}=w_{0}=\varphi_{x}=0\;\text{at}\;y=0,b;\;v_{0}=w_{0}=\varphi_{y}=0\;\text{at}\;x=0,a$$

The formal form of the solution satisfying the above boundary conditions is given as follows:

(52) $$u_{0}=\sum_{m}^{\infty }\sum_{n}^{\infty }U_{mn}\frac{\partial X_{m}\left(x\right)}{\partial x}Y_{n}\left(y\right)e^{-j\omega t}$$

(53) $$v_{0}=\sum_{m}^{\infty }\sum_{n}^{\infty }V_{mn}X_{m}\left(x\right)\frac{\partial Y_{n}\left(y\right)}{\partial y}e^{-j\omega t}$$

(54) $$w_{0}=\sum_{m}^{\infty }\sum_{n}^{\infty }W_{mn}X_{m}\left(x\right)Y_{n}\left(y\right)e^{-j\omega t}$$

(55) $$\varphi_{x}=\sum_{m}^{\infty }\sum_{n}^{\infty }\theta_{mn}^{x}\frac{\partial X_{m}\left(x\right)}{\partial x}Y_{n}\left(y\right)e^{-j\omega t}$$

(56) $$\varphi_{y}=\sum_{m}^{\infty }\sum_{n}^{\infty }\theta_{mn}^{y}X_{m}\left(x\right)\frac{\partial Y_{n}\left(y\right)}{\partial y}e^{-j\omega t}$$

where $j=\sqrt{-1}\text{,}$ $U_{mn},V_{mn},W_{mn},\theta_{mn}^{x}$ and $\theta_{mn}^{y}$ are unknown factors; $\omega =\omega_{mn}$ indicates the eigenfrequency related with the ${\left(m,n\right)}^{th}$ eigenmode. Under a certain assumption, eigenfunction $X_{m}\left(x\right)$ and $Y_{n}\left(y\right)$ can be constructed as in Table 1.

**Table 1:** Different boundary conditions are assigned to assumed eigenfunctions.

Boundary Condition	$X_{m}\left(x\right)$	$Y_{n}\left(y\right)$	Note
SSSS	$\sin\left(r_{m}x\right)$	$\sin\left(s_{n}y\right)$	$r_{m}=\frac{m\pi }{a}$; $s_{n}=\frac{n\pi }{b}$.
CCCC	$\sin^{2}\left(r_{m}x\right)$	$\sin^{2}\left(s_{n}y\right)$	
CCSS	$\sin\left(r_{m}x\right)\left(\cos\left(r_{m}x\right)-1\right)$	$\sin\left(s_{n}y\right)\left(\cos\left(s_{n}y\right)-1\right)$	

After performing a series of mathematical operations, we can get Eq. (57) by substituting Eqs. (52), (53), (54), (55), (56) into Eqs. (45), (46), (47), (48), (49), multiplying each equation by the relevant eigenfunction, and then integrating it over the solution's domain

(57) $$\left(K-\omega^{2}M\right)d=0\text{.}$$

in which K, M and d are the stiffness matrix, mass matrix, and displacement vector, respectively, which are expressed as follows (see Eqs. (58), (59)):

(58) $$K=\left[\begin{array}{ccccc} K_{mn}^{11} & K_{mn}^{12} & K_{mn}^{13} & K_{mn}^{14} & K_{mn}^{15}\\ K_{mn}^{12} & K_{mn}^{22} & K_{mn}^{23} & K_{mn}^{24} & K_{mn}^{25}\\ K_{mn}^{31} & K_{mn}^{32} & K_{mn}^{33} & K_{mn}^{34} & K_{mn}^{35}\\ K_{mn}^{41} & K_{mn}^{42} & K_{mn}^{43} & K_{mn}^{44} & K_{mn}^{45}\\ K_{mn}^{51} & K_{mn}^{52} & K_{mn}^{53} & K_{mn}^{54} & K_{mn}^{55} \end{array}\right];d=\left\{\begin{array}{c} U_{mn}\\ V_{mn}\\ W_{mn}\\ \theta_{mn}^{x}\\ \theta_{mn}^{y} \end{array}\right\}\text{.}$$

(59) $$M=\left[\begin{array}{ccccc} M_{mn}^{11} & 0 & 0 & M_{mn}^{14} & 0\\ 0 & M_{mn}^{22} & 0 & 0 & M_{mn}^{25}\\ 0 & 0 & M_{mn}^{33} & 0 & 0\\ M_{mn}^{41} & 0 & 0 & M_{mn}^{44} & 0\\ 0 & M_{mn}^{52} & 0 & 0 & M_{mn}^{55} \end{array}\right]\text{.}$$

in which $K_{mn}^{ij},M_{mn}^{ij}$ are the components of stiffness and mass matrices of fluid-infiltrated porous metal foam piezoelectric nanoplates and is figured out in the **Appendix**.

## 4 Numerical results

In this part, a set of Matlab 2018a calculation methods are established to evaluate the vibration response of a fluid-infiltrated porous metal foam piezoelectric nanoplate with variable thickness, nonlocal and flexoelectric influences, and Pasternak elastic foundation in a hygro-thermal environment. This set of programs is capable of evaluating variable thickness nanoplates under varied boundary conditions and has been independently checked for accuracy using confidence claims. The following non-dimensional representations are provided to facilitate comparisons between its numerical findings and those of other precise formulations provided elsewhere in the scientific literature:

$$\Omega_{1}=\omega_{1}a^{2}\sqrt{\rho t_{0}/D_{c}};{\bar{\Omega }}_{i}=\omega_{1}\frac{a^{2}}{\pi^{2}}\sqrt{\rho t_{0}/D_{c}}$$

$$K_{w}=\frac{k_{w}a^{4}}{D_{c}};K_{s}=\frac{k_{s}a^{2}}{D_{c}};\;D_{c}=\frac{E_{1}t_{0}^{3}}{12\left(1-v^{2}\right)}.\;f_{14}^{**}=10^{7}f_{014}\text{.}$$

### 4.1 Verifications

In this subsection, some numerical illustrations are carried out, and their results are compared with those available in the reference literature to verify the precision and reliability of the proposed approach. Firstly, the non-dimensional natural frequency $\Omega_{1}$ of the square isotropic plate with linear variable thickness in the *x*-axis in comparison with those of Kumar [64] using the Galerkin method according to FSDT and those of Manna [65] employing FEM based on FSDT are tabulated in Table 2. This table shows that the results of the current work are consistent with the results of Refs. [64,65]. However, when applying other boundary conditions such as CCCC or CCSS for plates with large thicknesses and tapers ($\gamma_{x})$, the results obtained are larger than those in the document [65], but the error range is still within the limit of 4%.

**Table 2:** Comparison of non-dimensional natural frequency $\Omega_{1}$ of the square isotropic plate with linear variable thickness in the *x*-axis.

$a/t_{0}$	Method	$\gamma_{x}$	SSSS	CCCC	CCSS
100	[64]	0.25	22.3079	41.9982	32.3085
	[65]		22.164	40.309	32.441
	Present		22.3080	40.7789	32.7197
	[64]	0.5	25.0594	–	–
	[65]		24.543	44.487	35.810
	Present		25.0595	45.7951	36.6635
	[64]	1.0	30.8965	–	–
	[65]		29.184	52.444	42.231
	Present		30.8968	56.4218	44.9332
10	[64]	0.25	21.3506	38.098	29.8564
	[65]		21.224	35.650	29.339
	Present		21.3597	37.0282	30.1800
	[64]	0.5	23.7276	–	–
	[65]		23.282	38.487	31.779
	Present		23.7401	40.6804	33.2047
	[64]	1.0	28.511	–	–
	[65]		27.12	43.410	36.069
	Present		28.5325	47.6477	39.0234
5	[64]	0.25	19.1493	30.976	25.043
	[65]		19.057	28.154	23.887
	Present		19.1749	30.1401	25.2334
	[64]	0.5	20.8337	–	–
	[65]		20.518	29.551	25.219
	Present		20.8664	32.0636	26.9725
	[64]	1.0	23.9282	–	–
	[65]		23.031	31.716	27.363
	Present		23.9772	35.2717	29.9518

Secondly, compare the non-dimensional natural frequency $\Omega_{1}$ of SSSS isotropic nanoplates with linearly varying *x*-axis thickness. The input data include nonlocal coefficient $\eta_{1}=0,1,2,4\;{nm}^{2}$; length-to-width ratio a/b=1,1.5,2 and taper $\gamma_{x}=0.4,0.8,1$. The frequency obtained is compared to thoset of Shahidi [66] which is shown in Table 3. From this table, it can be observed that the data of the proposed method are in good agreement with those of the document [66].

**Table 3:** Comparison of non-dimensional natural frequency $\Omega_{1}$ of SSSS isotropic nanoplate with linear variable thickness in the *x*-axis.

a/b	$\gamma_{x}$	Method	$\eta_{1}\left({nm}^{2}\right)$			
			0	1	2	4
1.0	0.4	[66]	23.6092	21.5725	19.9855	17.6407
		Present	23.8157	21.7643	20.1655	17.8028
	0.8	[66]	27.3629	24.9953	23.1509	20.4272
		Present	28.0736	25.6555	23.7709	20.9858
	1.0	[66]	29.2080	26.6764	24.7046	21.7934
		Present	30.2469	27.6415	25.6110	22.6103
2.0	0.4	[66]	58.7686	48.0194	41.5937	33.9620
		Present	58.8646	48.1671	41.7594	34.1336
	0.8	[66]	67.5375	55.0277	47.5806	38.7713
		Present	69.4528	56.8296	49.2689	40.2712
	1.0	[66]	71.7534	58.3704	50.4223	41.0417
		Present	74.8422	61.2384	53.0906	43.3946

Thirdly, An isotropic square plate clamped along its edges exposed to a uniform temperature rise above ambient temperature analyzed by P. Jeyaraj [67] is considered. They used a four noded plate element to predict the natural frequencies of the plate using FEM while the present work uses the analytical solution which is used to carry out the thermal vibration studies. The results obtained for a mild steel square plate (a=1m,h=0.01m) has been compared with P. Jeyaraj [67] as seen in Table 4 which shows first-two natural frequencies parameter $\Omega_{1},\Omega_{2}$. It can be seen that the results given by the article are completely consistent.

**Table 4:** Comparison of first –two natural frequencies $\Omega_{i}$ of CCCC isotropic plate with various temperature change.

	$\Omega_{1}$		$\Omega_{2}$	
ΔT	[67]	Present	[67]	Present
0	38	38.2844	78	78.2396
10	30	30.3262	69	69.3412
20	18	18.2454	58	58.7855

**Fig. 4:**
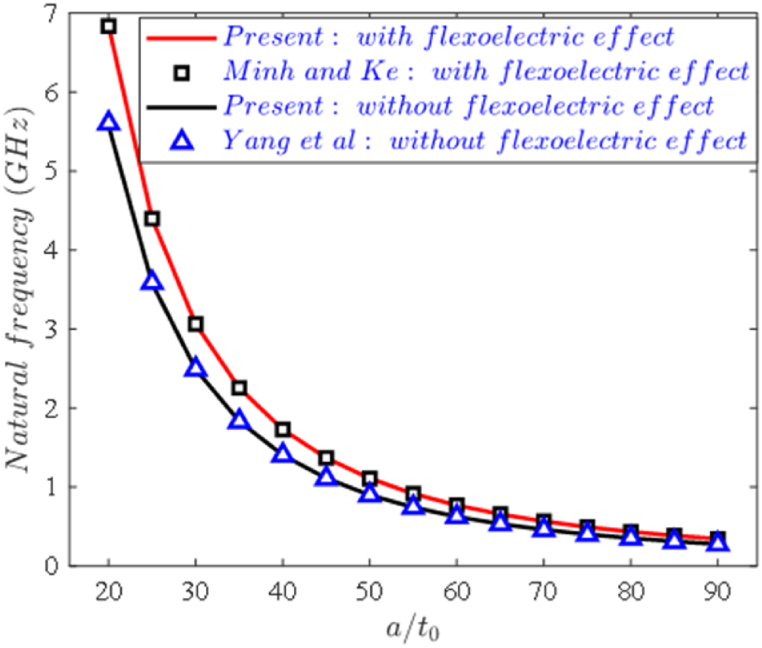
The natural frequency with the plate aspect ratio and flexoelectric effect.

Finally, the natural frequencies of SSSS piezoelectric nanoplates with geometric parameters $h=t_{0}=10nm$, and material properties $C_{11}=102GPa,C_{12}=31GPa,C_{66}=35.5GPa;e_{31}=-17.05C/m^{2},k_{33}=-1.76.10^{-8}\frac{C}{\text{Vm}},f_{14}^{**}=1\frac{C}{m}$ are taken into consideration. As seen in Fig. 4, these first natural frequencies for the piezoelectric nanoplate are compared to those of Minh and Ke [49] using FEM based on HSDT and those of Yang et al. [47] employing Navier approach based on CPT. Upon observing this figure, we can see that the results obtained in this study coincide with those of Yang et al. [47] (where the flexoelectric influence is not considered) and Minh and Ke [49] (where the flexoelectric effect is taken into account).

From the above comparison examples, it can be seen that the obtained results are excellently consistent with those of previous publications. Therefore, the proposed formulation for ensuring the trustworthiness and correctness of the next investigations.

### 4.2 Study parameters

The effects of the following parameters on the natural frequency of FPMF nanoplates are investigated in this section including: length-to-thickness ratio $a/t_{0}$; thickness parameter control $\gamma_{x},\gamma_{y},p_{x},p_{y}$; elastic foundation stiffnesses $K_{w}$ and $K_{s}$; temperature and moisture change, nonlocal coeffient; porosity coefficient; flexoelectric parameter; width-to-length ratio b/a; and boundary condition. PZT-5H has the following mechanical characteristics: $E_{1}=92.5784\;GPa,v=0.3039;e_{31}=-17.05C/m^{2},k_{33}=-1.76.10^{-8}\frac{C}{\text{Vm}},f_{14}^{**}=1\frac{C}{m}\text{.}$ Some input variables are set as follows: ${\;t}_{0}=10nm,a/t_{0}=20,b/a=1,\gamma_{x}=\gamma_{y}=0.2,p_{x}=p_{y}=1,\lambda_{0}=0.2,\eta_{1}=0.01a^{2}\;{nm}^{2},K_{w}=20,K_{s}=5,\alpha =0.3,\beta =0.3,\bar{\alpha }=10^{-5},\bar{\beta }=10^{-2},\Delta T=100,\Delta C=0.01$. (During the numerical investigation, certain parameters of the problem model will be varied, while the remaining parameters will remain as given above.)

Firstly, the first six natural frequencies (r, s = 11; 21; 12; 22; 31; 32) of variable thickness FPMF nanoplate with three different boundary conditions: CCCC, SSSS and CCSS are presented in Table 5-Table 8. The influence of the thickness control coefficients: $\gamma_{x},\gamma_{y},p_{x}\text{,}$ and $p_{y}$ on the dimensionless natural frequency of FPMF nanoplate is described in Table 5, Table 6. From these tables, it can be observed that the natural frequency of the plate increases with the growth of the coefficients $\gamma_{x},\gamma_{y}$. This is entirely consistent since as the coefficients $\gamma_{x},\gamma_{y}$ increase, so does the thickness of the plate, hence increasing its stiffness. In contrast to the rising of $\gamma_{x},\gamma_{y}$, the coefficients $p_{x},p_{y}$ increase, resulting in a drop in the natural frequency of the plate. The research considers three boundary conditions, among which the CCCC boundary makes the plate the strongest, but the SSSS boundary yields the lowest natural frequency. Finding a analytical solution for the free vibrations of the nanoplate with different boundary conditions is challenging due to the difficulty of establishing and solving mathematical equations. Nonetheless, achieving this goal is of significant mathematical and technological importance.

**Table 5:** Non-dimensional natural frequencies ${\bar{\Omega }}_{rs}$ of FPMF nanoplate with various parameter thickness control $\gamma_{x},\gamma_{y}$ and Type 1 porosity.

BCs	$\gamma_{x}$	$\gamma_{y}$	${\bar{\Omega }}_{11}$	${\bar{\Omega }}_{12}$	${\bar{\Omega }}_{21}$	${\bar{\Omega }}_{22}$	${\bar{\Omega }}_{31}$	${\bar{\Omega }}_{32}$
CCCC	0.25	0.25	4.9632	8.0132	9.3192	11.4159	11.6549	14.0050
		0.5	5.3117	8.6503	9.8304	12.1254	12.5144	14.6411
		1.0	5.9804	9.7912	10.8004	13.4296	13.9440	15.8175
	0.5	0.25	5.2992	8.6454	9.7944	12.1001	12.5725	14.5524
		0.5	5.6820	9.3199	10.3501	12.8527	13.4526	15.2310
		1.0	6.4040	10.5089	11.3839	14.2053	14.8974	16.4582
	1.0	0.25	5.9363	9.7849	10.6683	13.3438	14.1213	15.5062
		0.5	6.3736	10.5087	11.2906	14.1478	15.0161	16.2425
		1.0	7.1760	11.7499	12.4141	15.5373	16.4573	17.5301
SSSS	0.25	0.25	2.9736	5.7539	6.7446	8.9850	9.3089	11.3892
		0.5	3.1813	6.2431	7.1548	9.5981	10.0571	11.9739
		1.0	3.6059	7.1525	7.9631	10.7624	11.3301	13.0853
	0.5	0.25	3.1753	6.2385	7.1287	9.5752	10.0876	11.9006
		0.5	3.4120	6.7691	7.5834	10.2388	10.8736	12.5328
		1.0	3.8869	7.7411	8.4647	11.4734	12.1958	13.7132
	1.0	0.25	3.5832	7.1489	7.8596	10.6840	11.4705	12.8027
		0.5	3.8707	7.7446	8.3878	11.4206	12.3096	13.5047
		1.0	4.4328	8.8079	9.3891	12.7439	13.6970	14.7819
CCSS	0.25	0.25	3.8839	6.7851	8.0393	10.1694	10.3971	12.7335
		0.5	4.1711	7.3084	8.5192	10.8151	11.1511	13.3501
		1.0	4.7339	8.2672	9.4389	12.0181	12.4281	14.4913
	0.5	0.25	4.1501	7.3331	8.4989	10.8287	11.2527	13.3006
		0.5	4.4706	7.8945	9.0265	11.5216	12.0370	13.9627
		1.0	5.0895	8.9096	10.0187	12.7848	13.3519	15.1630
	1.0	0.25	4.6749	8.3457	9.3585	12.0494	12.7500	14.2940
		0.5	5.0517	8.9647	9.9583	12.8055	13.5713	15.0179
		1.0	5.7627	10.0593	11.0539	14.1335	14.9276	16.2903

**Table 6:** Non-dimensional natural frequencies ${\bar{\Omega }}_{rs}$ of FPMF nanoplate with various parameter thickness control $p_{x},p_{y}$ and Type 2 porosity.

BCs	$p_{x}$	$p_{y}$	${\bar{\Omega }}_{11}$	${\bar{\Omega }}_{12}$	${\bar{\Omega }}_{21}$	${\bar{\Omega }}_{22}$	${\bar{\Omega }}_{31}$	${\bar{\Omega }}_{32}$
CCCC	0.5	0.5	5.1229	8.3246	9.6144	11.7882	12.1224	14.4288
		1	5.0452	8.1726	9.5020	11.6293	11.8989	14.2921
		2	4.9569	7.9647	9.3786	11.4323	11.5780	14.1317
	1	0.5	5.0436	8.1748	9.4950	11.6260	11.9108	14.2775
		1	4.9680	8.0255	9.3854	11.4701	11.6896	14.1438
		2	4.8822	7.8215	9.2654	11.2770	11.3724	13.9304
	2	0.5	4.9488	7.9995	9.3319	11.4212	11.6575	14.0635
		1	4.8756	7.8533	9.2264	11.2691	11.4390	13.9341
		2	4.7927	7.6535	9.1110	11.0809	11.1262	13.6797
SSSS	0.5	0.5	3.0795	6.0042	6.9899	9.3088	9.7500	11.7959
		1	3.0337	5.8889	6.8975	9.1714	9.5538	11.6607
		2	2.9749	5.7341	6.7779	8.9959	9.2800	11.4877
	1	0.5	3.0331	5.8873	6.8957	9.1690	9.5542	11.6550
		1	2.9890	5.7748	6.8060	9.0349	9.3611	11.5233
		2	2.9322	5.6236	6.6900	8.8634	9.0920	11.3549
	2	0.5	2.9734	5.7358	6.7697	8.9904	9.3028	11.4603
		1	2.9313	5.6268	6.6838	8.8604	9.1138	11.3337
		2	2.8775	5.4807	6.5728	8.6944	8.8508	11.1719
CCSS	0.5	0.5	4.0148	7.0899	8.2958	10.5226	10.8759	13.1432
		1	3.9544	6.9456	8.1929	10.3670	10.6489	13.0013
		2	3.8786	6.7544	8.0642	10.1697	10.3378	12.8254
	1	0.5	3.9514	6.9569	8.1946	10.3762	10.6761	13.0037
		1	3.8929	6.8155	8.0943	10.2235	10.4523	12.8649
		2	3.8196	6.6284	7.9691	10.0292	10.1467	12.6922
	2	0.5	3.8756	6.7998	8.0555	10.1909	10.4337	12.8038
		1	3.8194	6.6619	7.9589	10.0418	10.2139	12.6696
		2	3.7490	6.4795	7.8385	9.8493	9.9170	12.4947

Table 7 illustrates the simultaneous effect of porosity coefficients $\lambda_{0}$ and Skempton β on the first six natural frequencies of the nanoplate. The statistics indicate that the natural frequency tends to grow when the coefficients $\beta ,\lambda_{0}$ are increased. This demonstrates that as the coefficient β rises, the plate becomes stiffer, but the change in frequency value induced by the coefficient β is minimal. An additional intriguing physical phenomenon is that there are no symmetric pairs of vibrations, despite the fact that the studied edges are symmetric, because the thickness of the plate varies according to its length and width positions, and the nonlocal and flexoelectric coefficients vary with respect to space.

**Table 7:** Non-dimensional natural frequencies ${\bar{\Omega }}_{rs}$ of FPMF nanoplate with various porosity and Skempton coefficient $\beta ,\lambda_{0}$ and Type 2 porosity ($p_{x}=0.5,p_{y}=2$).

BCs	β	$\lambda_{0}$	${\bar{\Omega }}_{11}$	${\bar{\Omega }}_{12}$	${\bar{\Omega }}_{21}$	${\bar{\Omega }}_{22}$	${\bar{\Omega }}_{31}$	${\bar{\Omega }}_{32}$
CCCC	0.1	0.1	4.8151	7.7103	9.0833	11.0512	11.1828	13.6422
		0.2	4.8396	7.7381	9.2018	11.1864	11.2488	13.8082
		0.4	4.8986	7.7792	9.4863	11.3545	11.4976	14.1736
	0.3	0.1	4.8584	7.7937	9.1483	11.1426	11.3055	13.7565
		0.2	4.8822	7.8215	9.2654	11.2770	11.3724	13.9304
		0.4	4.9398	7.8630	9.5469	11.4813	11.5863	14.2964
	0.5	0.1	4.8998	7.8731	9.2103	11.2296	11.4218	13.8364
		0.2	4.9229	7.9008	9.3262	11.3632	11.4895	14.0461
		0.4	4.9790	7.9424	9.6047	11.6013	11.6706	14.4126
SSSS	0.1	0.1	2.8803	5.5417	6.5509	8.6805	8.9385	11.0773
		0.2	2.9085	5.5619	6.6408	8.7889	8.9860	11.2810
		0.4	2.9834	5.5925	6.8589	9.0392	9.0531	11.7710
	0.3	0.1	2.9046	5.6036	6.6010	8.7557	9.0435	11.1524
		0.2	2.9322	5.6236	6.6900	8.8634	9.0920	11.3549
		0.4	3.0057	5.6542	6.9061	9.1126	9.1616	11.8420
	0.5	0.1	2.9280	5.6627	6.6490	8.8277	9.1433	11.2242
		0.2	2.9550	5.6826	6.7372	8.9347	9.1926	11.4255
		0.4	3.0270	5.7129	6.9512	9.1822	9.2648	11.9097
CCSS	0.1	0.1	3.7607	6.5322	7.8080	9.8254	9.9756	12.3948
		0.2	3.7865	6.5563	7.9122	9.9448	10.0331	12.6099
		0.4	3.8515	6.5927	8.1636	10.1045	10.2350	12.9561
	0.3	0.1	3.7943	6.6044	7.8660	9.9095	10.0892	12.4755
		0.2	3.8196	6.6284	7.9691	10.0292	10.1467	12.6922
		0.4	3.8832	6.6649	8.2180	10.2217	10.3175	13.0724
	0.5	0.1	3.8265	6.6732	7.9214	9.9895	10.1973	12.5523
		0.2	3.8512	6.6970	8.0235	10.1090	10.2552	12.7681
		0.4	3.9135	6.7335	8.2700	10.3312	10.3975	13.1828

Next, Table 8 depicts the simultaneous effect of nonlocal and flexoelectric coefficients on the first six natural frequencies of the FPMF nanoplate. It is evident that these two coefficients tend to have opposing effects, since the nonlocal coefficient reduces the natural frequency, resulting in a decrease in the stiffness of the plate when it is raised. In the other direction, when the flexoelectric coefficient rises, the plate's rigidity increases. Engineers and designers can compute the essential data sets to manage the vibrations of nanostructures by capturing this rising and decreasing pattern.

**Table 8:** Non-dimensional natural frequencies ${\bar{\Omega }}_{rs}$ of FPMF nanoplate with various flexoelectric and nonlocal coefficient $f_{14}^{**},\eta_{1}$ and Type 2 porosity ($p_{x}=0.5,p_{y}=0.5$).

BCs	$f_{14}^{**}$	$\sqrt{\eta_{1}/a}$	${\bar{\Omega }}_{11}$	${\bar{\Omega }}_{12}$	${\bar{\Omega }}_{21}$	${\bar{\Omega }}_{22}$	${\bar{\Omega }}_{31}$	${\bar{\Omega }}_{32}$
CCCC	0	0	4.8251	9.5290	9.5350	13.6876	16.3255	16.4063
		0.1	4.4096	7.8161	7.8222	10.3069	11.7476	11.7567
		0.2	3.6182	5.5766	5.5806	6.8555	7.5220	7.5976
	1	0	5.4421	9.7964	11.4521	15.2559	16.3057	19.6931
		0.1	4.9680	8.0255	9.3854	11.4701	11.6896	14.1438
		0.2	4.0639	5.7146	6.6725	7.5089	7.6013	8.8036
	2	0	6.7645	10.3727	14.8893	15.8736	18.4190	23.5943
		0.1	6.1699	8.4709	11.3243	12.2148	13.8387	15.5905
		0.2	5.0284	6.0004	7.2499	8.6693	9.1275	9.4596
SSSS	0	0	2.7823	6.6722	6.6788	10.3854	12.8166	12.8170
		0.1	2.5889	5.6082	5.6147	8.0478	9.4290	9.4294
		0.2	2.2019	4.1242	4.1290	5.4975	6.1635	6.1637
	1	0	3.2176	6.8696	8.1059	11.6731	12.7192	15.6735
		0.1	2.9890	5.7748	6.8060	9.0349	9.3611	11.5233
		0.2	2.5300	4.2445	4.9851	6.1208	6.1495	7.5108
	2	0	4.1564	7.3096	10.8673	12.2448	14.3816	18.2074
		0.1	3.8525	6.1452	9.0190	9.1197	11.1155	11.8044
		0.2	3.2409	4.5124	5.9036	6.6579	7.1703	7.5297
CCSS	0	0	3.7219	7.9865	8.1005	11.9862	14.4846	14.6477
		0.1	3.4249	6.6286	6.7194	9.1477	10.5264	10.6333
		0.2	2.8491	4.7974	4.8639	6.1598	6.8230	6.8906
	1	0	4.2343	8.2139	9.7644	13.4101	14.3883	17.7149
		0.1	3.8929	6.8155	8.0943	10.2235	10.4523	12.8649
		0.2	3.2290	4.9310	5.8380	6.7688	6.8664	8.1369
	2	0	5.3491	8.7212	12.8707	13.8980	16.3549	21.0009
		0.1	4.9126	7.2278	10.0760	10.6775	12.4606	13.4138
		0.2	4.0599	5.2151	6.5180	7.6866	8.0786	8.3294

**Fig. 5:**
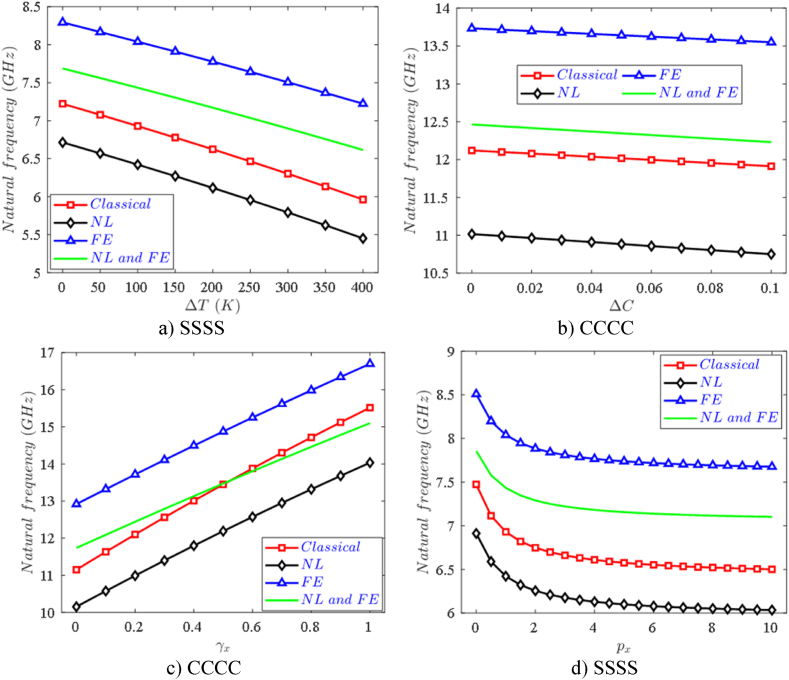
Effect of different theories on the first natural frequency of variable thickness FPMF nanoplate with Type 2 porosity.

Fig. 5 describes the influence of different theories on the first natural frequency (GHz) of the nanoplate, including classical plate theory, nonlocal theory, flexoelectric effect, and nonlocal theory combined with flexoelectric effect. The results indicate that the flexoelectric effect results in the highest natural frequency, whereas size-dependent effects contribute to the lowest natural frequency. Therefore, it can be seen that the flexoelectric effect makes the plate stiffer, however, size-dependent effects reduce the FPMF nanoplate stiffness. In addition, the results also show that when the FPMF nanoplate is in a multi-physical environment (affected by temperature and moisture), the stiffness of the plate decreases (Fig. 5a and b) as the temperature difference ΔT and moisture difference ΔC increases leading to the natural frequency will decrease. Fig. 5c and d also give an important finding for variable thickness FPMF nanoplates, as thickness control coefficients $\gamma_{x}$ and/or $\gamma_{y}$ increase, the natural frequency of nanoplates to increase, this is completely understandable because an increase in $\gamma_{x}$ and/or $\gamma_{y}$ make the nanoplate thicker (see Eq. (4)), so the plate be stiffer. In contrast, an increase in thickness control coefficients $p_{x}$ and/or $p_{y}$ causes the natural frequency of nanoplates to decrease, and the greatest decrease is when $0<p_{x}<2$, after which the rate of decrease of frequency gradually decreases. This research is pioneering when it gives natural frequencies for variable thickness nanostructures by analytical method.

**Fig. 6:**
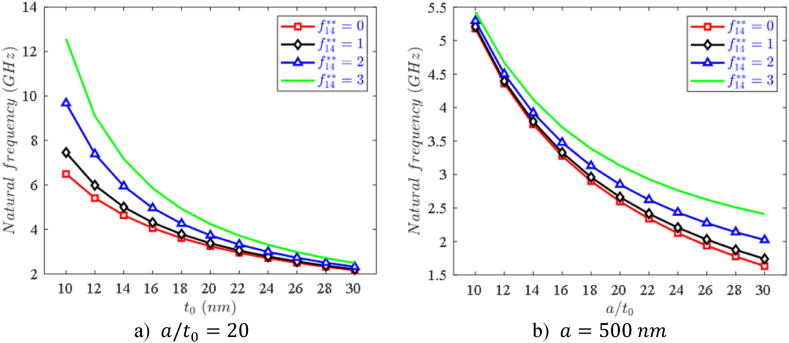
Effect of length-to-thickness and ratio $f_{14}^{**}$ on the first natural frequency of variable thickness FPMF nanoplate with Type 1 porosity and the SSSS boundary condition.

Next, the influence of length-to-thickness $a/t_{0}$ and $f_{14}^{**}$ on the first natural frequency of nanoplates is demonstrated in Fig. 6a and b. Flexoelectric coefficient $f_{14}^{**}$ is taken at 4 values 0, 1, 2, and 3. It can be observed that when the thickness $t_{0}$ is small, the difference in natural frequency due to the flexoelectric effect is very large, but when the thickness is larger, which indicates that the flexoelectric effect will fade away when the nanoplate becomes thicker. The results also show that $f_{14}^{**}$ makes the nanoplate stiffer, so the natural frequency is higher. This is explained by the fact that the $f_{14}^{**}$ coefficient appears directly in the hardness coefficients of piezoelectric materials (see Eq. (28a), (28b), (28c), (28d), (28e), (28f)), so the larger $f_{14}^{**}$ is, the more the material's hardness coefficient increases.

**Fig. 7:**
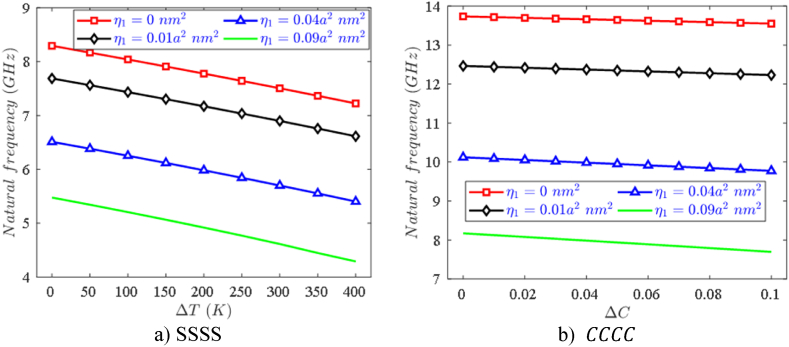
Effect of nonlocal coefficient, temperature ΔT and moisture ΔC on the first natural frequency of variable thickness FPMF nanoplates with Type 2 porosity.

Another important effect of nanostructures is the size-dependent effect, which is characterized by the nonlocal coefficient $\eta_{1}$, the first natural frequency results caused by this parameter can be observed in Fig. 7a and b. It is easy to see that the natural frequency of the plate decreases significantly as the nonlocal coefficient increases, thus, reducing the nanoplate stiffness can be controlled by increasing the nonlocal coefficient. Studies also show that, when the plate thickness becomes thinner than (a/h > 30), the impact of the nonlocal coefficient is no longer significant.

**Fig. 8:**
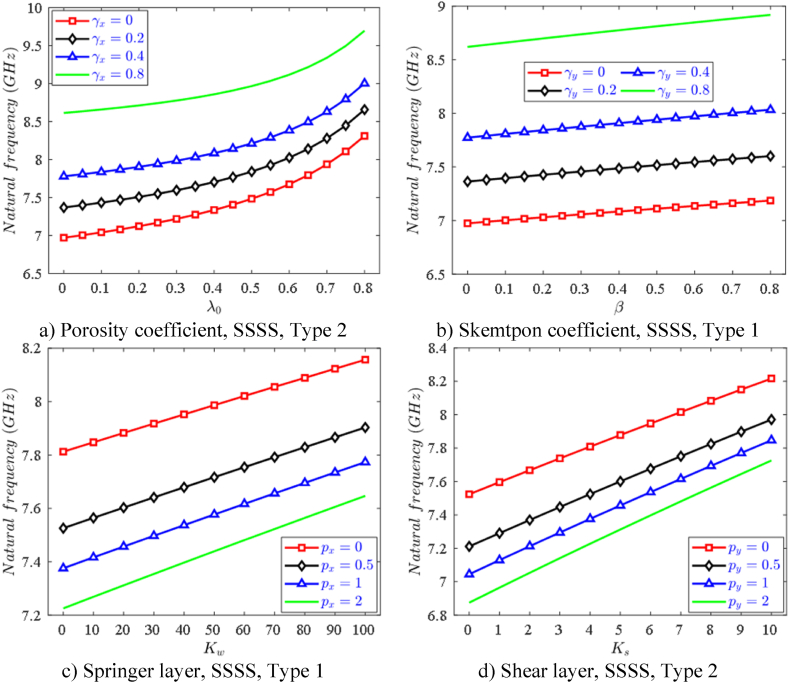
Effect of different parameters on the first natural frequency of variable FPMF thickness nanoplates.

Nowadays, macro to nanostructures with liquid-saturated micro-pores are becoming more and more popular with advantages such as being lighter, easier to adjust the mechanical properties of the structure, etc. Therefore, the impact of the porosity coefficient $\lambda_{0}$ (Fig. 8a) and Skempton factor $\bar{\beta }$ (Fig. 8b) on the first natural frequency of nanoplates is elucidated. It can be seen that both these parameters increase the first natural frequency of nanoplates, this does not mean that the pores make the plate stiffer, but the reason is explained here: the porous affect the whole elastic modulus *E* and mass density ρ and the interaction between stiffness and mass leads to an increase in the frequency of nanoplates. In addition, Fig. 8c and d also showed the effect of the foundation stiffness on increasing the natural frequency of FPMF nanoplates.

**Fig. 9:**
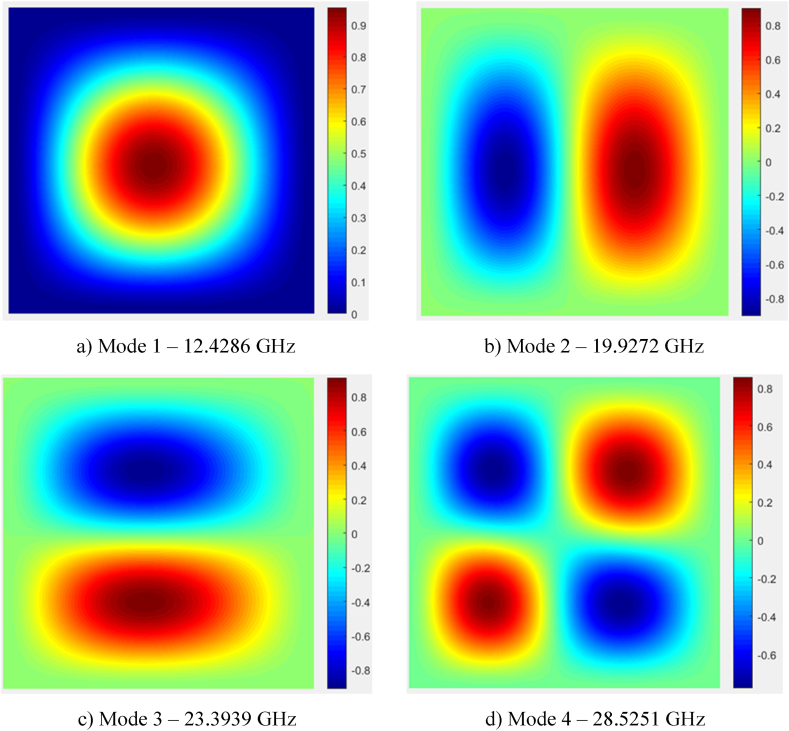
The first four vibration mode shape of the CCCC variable thickness FPMF nanoplate with Type 1 porosity.

Finally, Fig. 9a–d depicts four free vibration patterns of the CCCC/FPMF nanoplate. It is easy to see that, due to the varying thickness of the plates and the flexoelectric changes according to the thickness so that even if a symmetrical boundary condition is applied, the maximum and minimum oscillation points still deviate from symmetrical positions. These are also locations that can be used to place sensors during the measurement of structural parameters.

## 5 Conclusions

This paper utilizes the improved first-order shear plate theory and Galerkin-Vlasov method to investigate the hygro-thermal vibration of non-uniform thickness fluid-infiltrated porous metal foam piezoelectric nanoplates with flexoelectric and size-dependent effects. The thickness of the nanoplate changes according to a specified rule in both the length and width directions, while the nonlocal and flexoelectric coefficients vary with the material properties law along the thickness.

The results of this study demonstrate that the flexoelectric effect becomes more pronounced as the thickness of the nanoplate decreases. Additionally, increasing the nonlocal coefficients, temperature, and moisture can decrease the natural frequency, while increasing the porosity coefficient, Skempton factor, or elastic foundation stiffness can increase it. These parameters can be controlled by adjusting the thickness and configuration of the plate via control parameters. By shedding light on the size-dependent behavior of fluid-infiltrated porous metal foam piezoelectric nanostructures with variable thickness, this study has the potential to inform the development and application of innovative nanoplate-based sensors in NEMS. As this is the first endeavor on vibration studies of non-uniform thickness nanoplate with flexoelectric effect, the results provided in this manuscript can be used as a benchmark solution for future studies.

Some new points of this study:

The vibration response of fluid-infiltrated porous metal foam nanoplates.

The flexoelectricity and size small effects on the free vibration of nanoplates are considered.

The nanoplate's thickness varies in both the length and width directions.

The nonlocal and flexoelectric coefficients vary along the thickness and porous.

Analytical methods for calculating non-uniform thickness nanoplate under different boundary conditions.

## Data availability

Data used to support the findings of this study are included in the article.

## CRediT authorship contribution statement

**Thu-Huong Nguyen Thi:** Methodology, Investigation, Conceptualization. **Van Ke Tran:** Visualization, Software, Data curation. **Quoc Hoa Pham:** Writing – review & editing.

## Declaration of competing interest

The authors declare the following financial interests/personal relationships which may be considered as potential competing interests:Quoc Hoa Pham reports article publishing charges, statistical analysis, and writing assistance were provided by Nguyen Tat Thanh University. If there are other authors, they declare that they have no known competing financial interests or personal relationships that could have appeared to influence the work reported in this paper.

## Appendix

The coefficients of the stiffness matrix:

$$K_{mn}^{11}=\int_{0}^{b}\int_{0}^{a}\left(A_{11,x}X_{m,xx}Y_{n}+A_{11}X_{m,xxx}Y_{n}+A_{66,y}X_{m,x}Y_{n,y}+A_{66}X_{m,x}Y_{n,yy}\right)X_{m,x}Y_{n}dxdy\text{;}$$

$$K_{mn}^{12}=\int_{0}^{b}\int_{0}^{a}\left(A_{12,x}X_{m}Y_{n,yy}+A_{12}X_{m,x}Y_{n,yy}+A_{66,y}X_{m,x}Y_{n,y}+A_{66}X_{m,x}Y_{n,yy}\right)X_{m,x}Y_{n}dxdy\text{;}$$

$$K_{mn}^{13}=0;K_{mn}^{23}=0;\;K_{mn}^{31}=0;K_{mn}^{32}=0\text{;}$$

$$K_{mn}^{14}=\int_{0}^{b}\int_{0}^{a}\left(B_{11,x}X_{m,xx}Y_{n}+B_{11}X_{m,xxx}Y_{n}+B_{66,y}X_{m,x}Y_{n,y}+B_{66}X_{m,x}Y_{n,yy}\right)X_{m,x}Y_{n}dxdy$$

$$K_{mn}^{15}=\int_{0}^{b}\int_{0}^{a}\left(B_{12,x}X_{m}Y_{n,yy}+B_{12}X_{m,x}Y_{n,yy}+B_{66,y}X_{m,x}Y_{n,y}+B_{66}X_{m,x}Y_{n,yy}\right)X_{m,x}Y_{n}dxdy$$

$$K_{mn}^{21}=\int_{0}^{b}\int_{0}^{a}\left(A_{12,y}X_{m,xx}Y_{n}+A_{12}X_{m,xx}Y_{n,y}+A_{66,x}X_{m,x}Y_{n,y}+A_{66}X_{m,xx}Y_{n,y}\right)X_{m}Y_{n,y}dxdy\text{;}$$

$$K_{mn}^{22}=\int_{0}^{b}\int_{0}^{a}\left(A_{22,y}X_{m}Y_{n,yy}+A_{22}X_{m}Y_{n,yyy}+A_{66,x}X_{m,x}Y_{n,y}+A_{66}X_{m,xx}Y_{n,y}\right)X_{m}Y_{n,y}dxdy\text{;}$$

$$K_{mn}^{24}=\int_{0}^{b}\int_{0}^{a}\left(B_{12,y}X_{m,xx}Y_{n}+B_{12}X_{m,xx}Y_{n,y}+B_{66,x}X_{m,x}Y_{n,y}+B_{66}X_{m,xx}Y_{n,y}\right)X_{m}Y_{n,y}dxdy\text{;}$$

$$K_{mn}^{25}=\int_{0}^{b}\int_{0}^{a}\left(B_{22,y}X_{m}Y_{n,yy}+B_{22}X_{m}Y_{n,yyy}+B_{66,x}X_{m,x}Y_{n,y}+B_{66}X_{m,xx}Y_{n,y}\right)X_{m}Y_{n,y}dxdy\text{;}$$

$$K_{mn}^{33}=\int_{0}^{b}\int_{0}^{a}\left(A_{55,x}^{s}X_{m,x}Y_{n}+A_{55}^{s}X_{m,xx}Y_{n}+A_{44,y}^{s}X_{m}Y_{n,y}+A_{44}^{s}X_{m}Y_{n,yy}-k_{w}X_{m}Y_{n}+\left(k_{w\mu }+k_{s}+N^{0}+N^{T}+N^{C}\right)\left(X_{m,xx}Y_{n}+X_{m}Y_{n,yy}\right)-\left(k_{s\mu }+N^{\mu 0}+N^{\mu T}+N^{\mu C}\right)\left(X_{m,xxxx}Y_{n}+X_{m}Y_{n,yyyy}+2X_{m,xx}Y_{n,yy}\right)-\left(N_{,xx}^{\mu T}+N_{,xx}^{\mu C}+N_{,yy}^{\mu T}+N_{,yy}^{\mu C}\right)\left(X_{m,xx}Y_{n}+X_{m}Y_{n,yy}\right)-2\left(N_{,x}^{\mu T}+N_{,x}^{\mu C}\right)\left(X_{m,xxx}Y_{n}+X_{m,x}Y_{n,yy}\right)-2\left(N_{,y}^{\mu T}+N_{,y}^{\mu C}\right)\left(X_{m,xx}Y_{n,y}+X_{m}Y_{n,yyy}\right)\right)X_{m}Y_{n}dxdy\text{;}$$

$$K_{mn}^{34}=\int_{0}^{b}\int_{0}^{a}\left(A_{55,x}^{s}X_{m,x}Y_{n}+A_{55}^{s}X_{m,xx}Y_{n}\right)X_{m}Y_{n}dxdy\text{;}$$

$$K_{mn}^{35}=\int_{0}^{b}\int_{0}^{a}\left(A_{44,y}^{s}X_{m}Y_{n,y}+A_{44}^{s}X_{m}Y_{n,yy}\right)X_{m}Y_{n}dxdy\text{;}$$

$$K_{mn}^{41}=\int_{0}^{b}\int_{0}^{a}\left(\left({\bar{B}}_{11,x}+S_{11,x}\right)X_{m,xx}Y_{n}+\left({\bar{B}}_{11}+S_{11}\right)X_{m,xxx}Y_{n}+{\bar{B}}_{66,y}X_{m,x}Y_{n,y}+{\bar{B}}_{66}X_{m,x}Y_{n,yy}\right)X_{m,x}Y_{n}dxdy\text{;}$$

$$K_{mn}^{42}=\int_{0}^{b}\int_{0}^{a}\left(\left({\bar{B}}_{12,x}+S_{11,x}\right)X_{m}Y_{n,yy}+\left({\bar{B}}_{12}+S_{11}\right)X_{m,x}Y_{n,yy}+{\bar{B}}_{66,y}X_{m,x}Y_{n,y}+{\bar{B}}_{66}X_{m,x}Y_{n,yy}\right)X_{m,x}Y_{n}dxdy\text{;}$$

$$K_{mn}^{43}=-\int_{0}^{b}\int_{0}^{a}\left(A_{55}^{s}X_{m,x}Y_{n}\right)X_{m,x}Y_{n}dxdy\text{;}$$

$$K_{mn}^{44}=\int_{0}^{b}\int_{0}^{a}\left(\left(F_{11,x}+S_{12,x}\right)X_{m,xx}Y_{n}+\left(F_{11}+S_{12}\right)X_{m,xxx}Y_{n}+F_{66,y}X_{m,x}Y_{n,y}+F_{66}X_{m,x}Y_{n,yy}-A_{55}^{s}X_{m,x}Y_{n}\right)X_{m,x}Y_{n}dxdy$$

$$K_{mn}^{45}=\int_{0}^{b}\int_{0}^{a}\left(\left(F_{12,x}+S_{12,x}\right)X_{m}Y_{n,yy}+\left(F_{12}+S_{12}\right)X_{m,x}Y_{n,yy}+F_{66,y}X_{m,x}Y_{n,y}+F_{66}X_{m,x}Y_{n,yy}\right)X_{m,x}Y_{n}dxdy$$

$$K_{mn}^{51}=\int_{0}^{b}\int_{0}^{a}\left(\left({\bar{B}}_{12,y}+S_{11,y}\right)X_{m,xx}Y_{n}+\left({\bar{B}}_{12}+S_{11}\right)X_{m,xx}Y_{n,y}+{\bar{B}}_{66,x}X_{m,x}Y_{n,y}+{\bar{B}}_{66}X_{m,xx}Y_{n,y}\right)X_{m}Y_{n,y}dxdy\text{;}$$

$$K_{mn}^{52}=\int_{0}^{b}\int_{0}^{a}\left(\left({\bar{B}}_{22,y}+S_{11,y}\right)X_{m}Y_{n,yy}+\left({\bar{B}}_{22}+S_{11}\right)X_{m}Y_{n,yyy}+{\bar{B}}_{66,x}X_{m,x}Y_{n,y}+{\bar{B}}_{66}X_{m,xx}Y_{n,y}\right)X_{m}Y_{n,y}dxdy\text{;}$$

$$K_{mn}^{53}=-\int_{0}^{b}\int_{0}^{a}\left(A_{44}^{s}X_{m}Y_{n,y}\right)X_{m}Y_{n,y}dxdy\text{;}$$

$$K_{mn}^{54}=\int_{0}^{b}\int_{0}^{a}\left(\left(F_{12,y}+S_{12,y}\right)X_{m,xx}Y_{n}+\left(F_{12}+S_{12}\right)X_{m,xx}Y_{n,y}+F_{66,x}X_{m,x}Y_{n,y}+F_{66}X_{m,xx}Y_{n,y}\right)X_{m}Y_{n,y}dxdy\text{;}$$

$$K_{mn}^{55}=\int_{0}^{b}\int_{0}^{a}\left(\left(F_{22,y}+S_{12,y}\right)X_{m}Y_{n,yy}+\left(F_{22}+S_{12}\right)X_{m}Y_{n,yyy}+F_{66,x}X_{m,x}Y_{n,y}+F_{66}X_{m,xx}Y_{n,y}-A_{44}^{s}X_{m}Y_{n,y}\right)X_{m}Y_{n,y}dxdy\text{;}$$

The coefficients of the mass matrix:

$$M_{mn}^{11}=\int_{0}^{b}\int_{0}^{a}\left(I_{0}X_{m,x}Y_{n}-J_{0,xx}X_{m,x}Y_{n}-2J_{0,x}X_{m,xx}Y_{n}-J_{0}X_{m,xxx}Y_{n}-J_{0,yy}X_{m,x}Y_{n}-2J_{0,y}X_{m,x}Y_{n,y}-J_{0}X_{m,x}Y_{n,yy}\right)X_{m,x}Y_{n}dxdy$$

$$M_{mn}^{14}=\int_{0}^{b}\int_{0}^{a}\left(I_{1}X_{m,x}Y_{n}-J_{1,xx}X_{m,x}Y_{n}-2J_{1,x}X_{m,xx}Y_{n}-J_{1}X_{m,xxx}Y_{n}-J_{1,yy}X_{m,x}Y_{n}-2J_{1,y}X_{m,x}Y_{n,y}-J_{1}X_{m,x}Y_{n,yy}\right)X_{m,x}Y_{n}dxdy$$

$$M_{mn}^{22}=\int_{0}^{b}\int_{0}^{a}\left(I_{0}X_{m}Y_{n,y}-J_{0,xx}X_{m}Y_{n,y}-2J_{0,x}X_{m,x}Y_{n,y}-J_{0}X_{m,xx}Y_{n,y}-J_{0,yy}X_{m}Y_{n,y}-2J_{0,y}X_{m}Y_{n,yy}-J_{0}X_{m}Y_{n,yyy}\right)X_{m}Y_{n,y}dxdy\text{;}$$

$$M_{mn}^{25}=\int_{0}^{b}\int_{0}^{a}\left(I_{1}X_{m}Y_{n,y}-J_{1,xx}X_{m}Y_{n,y}-2J_{1,x}X_{m,x}Y_{n,y}-J_{1}X_{m,xx}Y_{n,y}-J_{1,yy}X_{m}Y_{n,y}-2J_{1,y}X_{m}Y_{n,yy}-J_{1}X_{m}Y_{n,yyy}\right)X_{m}Y_{n,y}dxdy\text{;}$$

$$M_{mn}^{33}=\int_{0}^{b}\int_{0}^{a}\left(I_{0}X_{m}Y_{n}-J_{0,xx}X_{m}Y_{n}-2J_{0,x}X_{m,x}Y_{n}-J_{0}X_{m,xx}Y_{n}-J_{0,yy}X_{m}Y_{n}-2J_{0,y}X_{m}Y_{n,y}-J_{0}X_{m}Y_{n,yy}\right)X_{m}Y_{n}dxdy\text{;}$$

$$M_{mn}^{41}=\int_{0}^{b}\int_{0}^{a}\left(I_{1}X_{m,x}Y_{n}-J_{1,xx}X_{m,x}Y_{n}-2J_{1,x}X_{m,xx}Y_{n}-J_{1}X_{m,xxx}Y_{n}-J_{1,yy}X_{m,x}Y_{n}-2J_{1,y}X_{m,x}Y_{n,y}-J_{1}X_{m,x}Y_{n,yy}\right)X_{m,x}Y_{n}dxdy\text{;}$$

$$M_{mn}^{44}=\int_{0}^{b}\int_{0}^{a}\left(I_{2}X_{m,x}Y_{n}-J_{2,xx}X_{m,x}Y_{n}-2J_{2,x}X_{m,xx}Y_{n}-J_{2}X_{m,xxx}Y_{n}-J_{2,yy}X_{m,x}Y_{n}-2J_{2,y}X_{m,x}Y_{n,y}-J_{2}X_{m,x}Y_{n,yy}\right)X_{m,x}Y_{n}dxdy\text{;}$$

$$M_{mn}^{52}=\int_{0}^{b}\int_{0}^{a}\left(I_{1}X_{m}Y_{n,y}-J_{1,xx}X_{m}Y_{n,y}-2J_{1,x}X_{m,x}Y_{n,y}-J_{1}X_{m,xx}Y_{n,y}-J_{1,yy}X_{m}Y_{n,y}-2J_{1,y}X_{m}Y_{n,yy}-J_{1}X_{m}Y_{n,yyy}\right)X_{m}Y_{n,y}dxdy\text{;}$$

$$M_{mn}^{55}=\int_{0}^{b}\int_{0}^{a}\left(I_{2}X_{m}Y_{n,y}-J_{2,xx}X_{m}Y_{n,y}-2J_{2,x}X_{m,x}Y_{n,y}-J_{2}X_{m,xx}Y_{n,y}-J_{2,yy}X_{m}Y_{n,y}-2J_{2,y}X_{m}Y_{n,yy}-J_{2}X_{m}Y_{n,yyy}\right)X_{m}Y_{n,y}dxdy\text{;}$$
